# Optimization of ribosome profiling in plants including structural analysis of rRNA fragments

**DOI:** 10.1186/s13007-024-01267-3

**Published:** 2024-09-16

**Authors:** Michael K. Y. Ting, Yang Gao, Rouhollah Barahimipour, Rabea Ghandour, Jinghan Liu, Federico Martinez-Seidel, Julia Smirnova, Vincent Leon Gotsmann, Axel Fischer, Michael J. Haydon, Felix Willmund, Reimo Zoschke

**Affiliations:** 1 Max Planck Institute of Molecular Plant Physiology, Am Mühlenberg 1, 14476 Potsdam-Golm, Germany; 2 Technical University Kaiserslautern, Paul-Ehrlich-Str. 23, 67663 Kaiserslautern, Germany; 3 School of BioSciences, University of Melbourne, VIC, Melbourne, 3010 Australia; 4 Charité Universitätsmedizin, Charitéplatz 1, 10117 Berlin, Germany; 5 Universität Marburg, Karl-von-Frisch-Str. 14, 35032 Marburg, Germany

**Keywords:** *Arabidopsis thaliana*, *Nicotiana tabacum*, Translation, Ribosome profiling, Ribo-seq, Guidelines

## Abstract

**Background:**

Ribosome profiling (or Ribo-seq) is a technique that provides genome-wide information on the translational landscape (translatome). Across different plant studies, variable methodological setups have been described which raises questions about the general comparability of data that were generated from diverging methodologies. Furthermore, a common problem when performing Ribo-seq are abundant rRNA fragments that are wastefully incorporated into the libraries and dramatically reduce sequencing depth. To remove these rRNA contaminants, it is common to perform preliminary trials to identify these fragments because they are thought to vary depending on nuclease treatment, tissue source, and plant species.

**Results:**

Here, we compile valuable insights gathered over years of generating Ribo-seq datasets from different species and experimental setups. We highlight which technical steps are important for maintaining cross experiment comparability and describe a highly efficient approach for rRNA removal. Furthermore, we provide evidence that many rRNA fragments are structurally preserved over diverse nuclease regimes, as well as across plant species. Using a recently published cryo-electron microscopy (cryo-EM) structure of the tobacco 80S ribosome, we show that the most abundant rRNA fragments are spatially derived from the solvent-exposed surface of the ribosome.

**Conclusion:**

The guidelines presented here shall aid newcomers in establishing ribosome profiling in new plant species and provide insights that will help in customizing the methodology for individual research goals.

**Supplementary Information:**

The online version contains supplementary material available at 10.1186/s13007-024-01267-3.

## Introduction

Ribosome profiling was first described in 2009 [23] and since then has revolutionized our understanding of translation by providing genome-wide information about ribosome occupancy within translated regions. The method uses a ribonuclease treatment to degrade regions of mRNAs that are not protected by translating ribosomes. The remaining ribosome protected fragments (RPFs also called ribosome footprints) can be purified and examined by deep sequencing, which provides genome-wide information on the translational landscape. In addition, the position of the ribosome peptidyl site (P-site) can be computationally estimated for each RPF, thereby providing codon-level resolution of the translation activity. The original ribosome profiling technique has continually been improved, and it is often fine-tuned for individual species and tissues. Examples of recent and comprehensive descriptions of this technique are available for yeast and mice [18], human and drosophila [13], and bacteria [36]. In plants, translatomes have been assessed in several species including Arabidopsis (*Arabidopsis thaliana*) [4, 21, 24, 30, 31, 35], maize [8, 27], tomato [5, 48], and rice [51, 52]. Since plant chloroplasts encode a distinct set of prokaryote-like ribosomes, specialized protocols also exist for assessing the chloroplast translatome [7, 17, 42, 56].

With the growing number of plant ribosome profiling studies, methodological variations have arisen, which might introduce potential biases at several steps [1]. *Extraction buffer composition*: the ionic strength and buffering capacity of the extraction buffer can affect the observed behavior of RPFs [21]. *Choice of ribonuclease*: several ribonucleases have been used for the generation of RPFs, including RNase I, A, T1 and MNase [18]. Some ribonucleases exhibit preferential cleavage at specific motifs, thereby confounding codon resolution. To date, the most widely used ribonuclease for Ribo-seq in eukaryotes is RNase I [22], whereas MNase is the preferred ribonuclease for Ribo-seq in prokaryotes [36]. *Ribonuclease treatment*: the amount of ribonuclease, digestion time, and digestion temperature can vary across studies. In addition, ribonuclease treatment can be performed directly on cell lysates or on purified polysomes. *RPF purification strategy*: some protocols capture RPFs within a narrow size range (e.g., 28–30 nt), which enriches the highly periodic RPFs [21]. Others prefer to use a broader size range (e.g., 20–40 nt), which also has notable benefits [8]. Importantly, a broader size range is inclusive of unique RPFs that convey valuable information about translational dynamics, such as the 21 nt RPFs that represent ribosomes lacking a tRNA in the A-site [47]. *rRNA removal*: since ribosomes are composed of RNA, ribonuclease treatment unavoidably leads to the generation of widespread nicks in rRNA, creating fragments which can co-purify with RPFs. These unwanted rRNA fragments are wastefully incorporated into the sequencing libraries and substantially reduce the number of informative reads. Small scale sequencing tests are often performed to identify the major fragments from individual experimental setups [32]. Enzymatic strategies to remove rRNA have been described [10] but these methods have been shown to perturb codon-resolution [54]. The most commonly applied approach to remove rRNA contamination is subtractive hybridization using biotinylated DNA oligonucleotides (oligos). *Library preparation*: the original ribosome profiling method used RNA circularization to incorporate RPFs into a cDNA library, which is a method still used by many labs. Libraries can also be prepared from kits designed for sequencing of small-RNA that utilize RNA ligases for adapter incorporation [8], as well as ligation-free approaches that utilize polyadenylation and reverse transcription template-switching [20].

Such methodological variation can seem overwhelming to those performing ribosome profiling for the first time, and/or to those who wish to establish the technique in a new plant species. It also raises concerns of the comparability of datasets across different studies that have utilized different methodologies. Here, we focus on data reproducibility by compiling valuable insights gathered over years of generating Ribo-seq datasets from different plant species and experimental setups. We also provide a structural analysis of the rRNA fragments that regularly contaminate Ribo-seq libraries, and reveal patterns that are spatially preserved over diverse nuclease treatments, as well as across plant species. Overall, these guidelines are anticipated to be a valuable resource for the plant community and should be applicable to any Ribo-seq methodology.

## Materials and methods

The following section provides information for the samples, which were prepared over different stages of protocol optimization. Thus, the data presented are derived from different plant material from diverse experiments. The detailed, fully optimized protocol is provided in the Supplemental Methods.

### Plant material

**Fig. 1:**
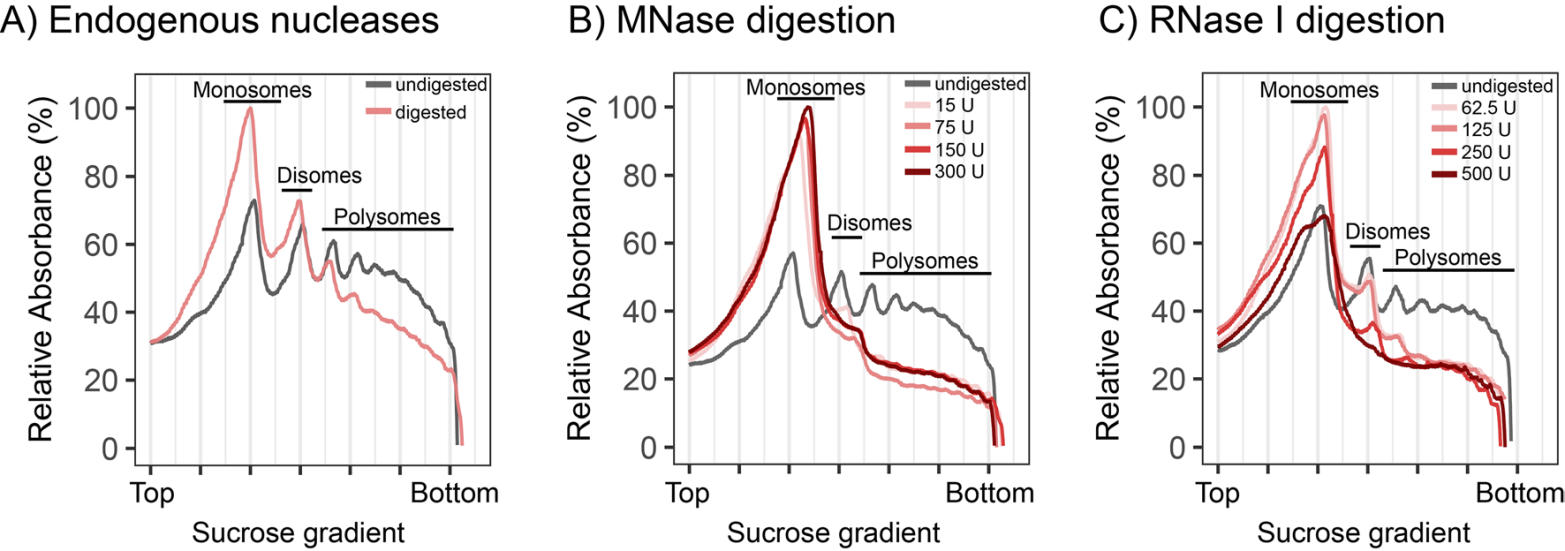
Qualitative assessment of ribonuclease concentrations by polysome profiling. Polysome profiling was used to determine optimal nuclease concentrations for converting polysomes into monosomes. **(A-C)** Sucrose gradient profiles of ribonucleoprotein particles derived from wild-type tobacco leaf lysates, following treatment with **(A)** Endogenous nucleases, or endogenous nucleases supplemented with **(B)** MNase or **(C)** RNase I. Undigested samples contain the ribonuclease inhibitor heparin to inhibit nuclease activity. Ribonucleoprotein particles were size-separated in sucrose density gradients (15, 30, 45, and 60% w/v from top to bottom) by ultracentrifugation as previously described [16]. The signals of monosomes, disomes and polysomes were detected by UV absorbance measurements (254 nm). U, Units of applied ribonuclease (note that the specified RNase activity was applied per 1 mL of plant lysate, derived from 100 mg of plant fresh weight tissue)

The tissue used for the comparison of RNase I and MNase digestion, was derived from 8-day old Arabidopsis seedlings (Col-0) grown on ½ Murashige and Skoog medium [37] with 6.8% agar and 1% sucrose, grown at 100 µmol m^− 2^s^− 1^ for 16 h/8 h light/dark cycles at 20 °C. The tissue used for refining RNase I treatment, rRNA depletion and comparison of ligation-free to ligation-based strategies, were derived from 14-day old Arabidopsis seedlings (Col-0), grown on ½ Murashige and Skoog media with 1% agar, grown at 100 µmol m^− 2^s^− 1^ for 12 h/12 h light/dark cycles at 20 °C. Tobacco (*Nicotiana tabacum*) tissue used for ribosome profiling was derived from a temperature-shift experiment, from leaves harvested from 28-day old plants grown on soil at 350 µmol m^− 2^s^− 1^ in 16 h/8 h light/dark cycles at 12 °C. Tobacco (*Nicotiana tabacum*) tissue used for polysome profiling (Fig. 1) was derived from leaves harvested from 21-day old plants grown on soil at 350 µmol m^− 2^s^− 1^ in 16 h/8 h light/dark cycles at 24 °C.

### RNA and RPF isolation

Total RNA and RPFs were isolated as previously described [46] with modifications described in the Supplemental Methods. The units (U) of ribonuclease used in this study are normalized to one mL of plant lysate, derived from 100 mg of plant fresh weight. Since Ca^2+^ is a known cofactor of MNase, samples digested with MNase include 5 mM CaCl_2_. All RPFs that were not rRNA-depleted were size-selected between 20 and 50 nt. All rRNA-depleted RPFs were size-selected between 20 and 35 nt. Details of rRNA depletion are available in the supplemental methods.

### Library preparation

For the ligation-free strategy, rRNA depleted RPFs were directly used as input for the D-plex small RNA-seq kit (Diagenode cat#C05030001), according to manufacturer’s instructions. Diagenode libraries are typically amplified with 7–9 PCR cycles. For the RNA-ligase strategy, the terminal ends of the RPFs were first repaired using T4 polynucleotide kinase (PNK; ThermoFisher, cat#EK0031). This was carried out in 20 µL volume with ~ 100 ng of RPFs (un-depleted) or ~ 30 ng of RPFs (rRNA-depleted), as described in the supplemental methods. After treatment, RPFs were directly used as input into the NEXTflex small RNA-seq kit v3 (Perkin Elmer, cat# NOVA-5132-06) or V4 (Perkin Elmer, cat#NOVA-5132-31), according to the manufacturer’s instructions. NEXTflex libraries are typically amplified using 14–16 PCR cycles. Libraries were sequenced on a Nextseq500 (SE75) or Novaseq6000 (SE100). The sequencing data have been deposited in NCBI’s Gene Expression Omnibus under accession number GSE226508.

### Identification of major rRNA fragments

To identify the most abundant rRNA fragments, pioneer Ribo-seq libraries were aligned to rRNA genes as described in the Supplemental methods. Each rRNA gene was then visually inspected in the IGV browser (http://software.broadinstitute.org/software/igv) to identify regions with high coverage that were repeatedly present in the majority of the libraries. Complementary biotinylated DNA oligos were designed (Table S1 and S2) and mixed together in molar ratios equivalent to the relative averaged abundance of the target rRNA contaminant within these pioneer libraries.

### Mapping rRNA fragments to the ribosome structure

The reference structure used in this work corresponds to the translating cytosolic ribosome of *Nicotiana tabacum* (PDB: 8B2L, EMDB: 15806) [45]. The structure was solved by using single-particle cryo-electron microscopy to an overall resolution of 2.2 Å. The molecular model of the tobacco 80S ribosome contains in total 91% of the rRNA residues within the small and 95% of the rRNA residues within the large ribosomal subunit. The top contaminating rRNA fragments, derived from pioneer tobacco Ribo-seq datasets, were mapped to the 80S ribosome model using PyMOL (The PyMOL Molecular Graphics System, Version 1.2r3pre, Schrödinger, LLC) and colored according to their relative abundance.

## Results and discussion

### Effects of variable nuclease treatments

In the early stages of establishing plant ribosome profiling in our group, we were initially concerned that RPFs generated using different nucleases and/or nuclease concentrations, might lead to technical variation that limits reproducibility. For example, not using sufficient nuclease (under digestion) could become problematic if there exists a population of ribosomes that preferentially remained in the polysome fraction (i.e., some transcripts that are more resistant to nuclease digestion due to RNA binding proteins or RNA secondary structure). Such a bias would result in reduced RPF yield, and/or alter the quantitative translatome. On the other hand, using excessive nuclease (over digestion) was anticipated to increase rRNA fragmentation, overly breaking down ribosomes and subsequently reducing RPF yield. To address these concerns, polysome profiling was performed to identify the minimal nuclease concentration required to efficiently convert polysomes into monosomes, without causing excessive monosome breakdown. When performing digestion directly on cell lysate, endogenous nuclease activity also contributes to monosome formation (Fig. 1A). All MNase concentrations tested produced similar profiles, highlighting the general robustness of treatments with this nuclease (Fig. 1B). For RNase I, disomes and higher order polysomes remained visible from samples treated with less than 250 U, suggestive of under digestion, whereas using more than 250 U resulted in monosome reduction, suggestive of over digestion (Fig. 1C; note that all RNase unit specifications are given per 1 mL of plant lysate, derived from 100 mg of plant fresh weight).

**Fig. 2:**
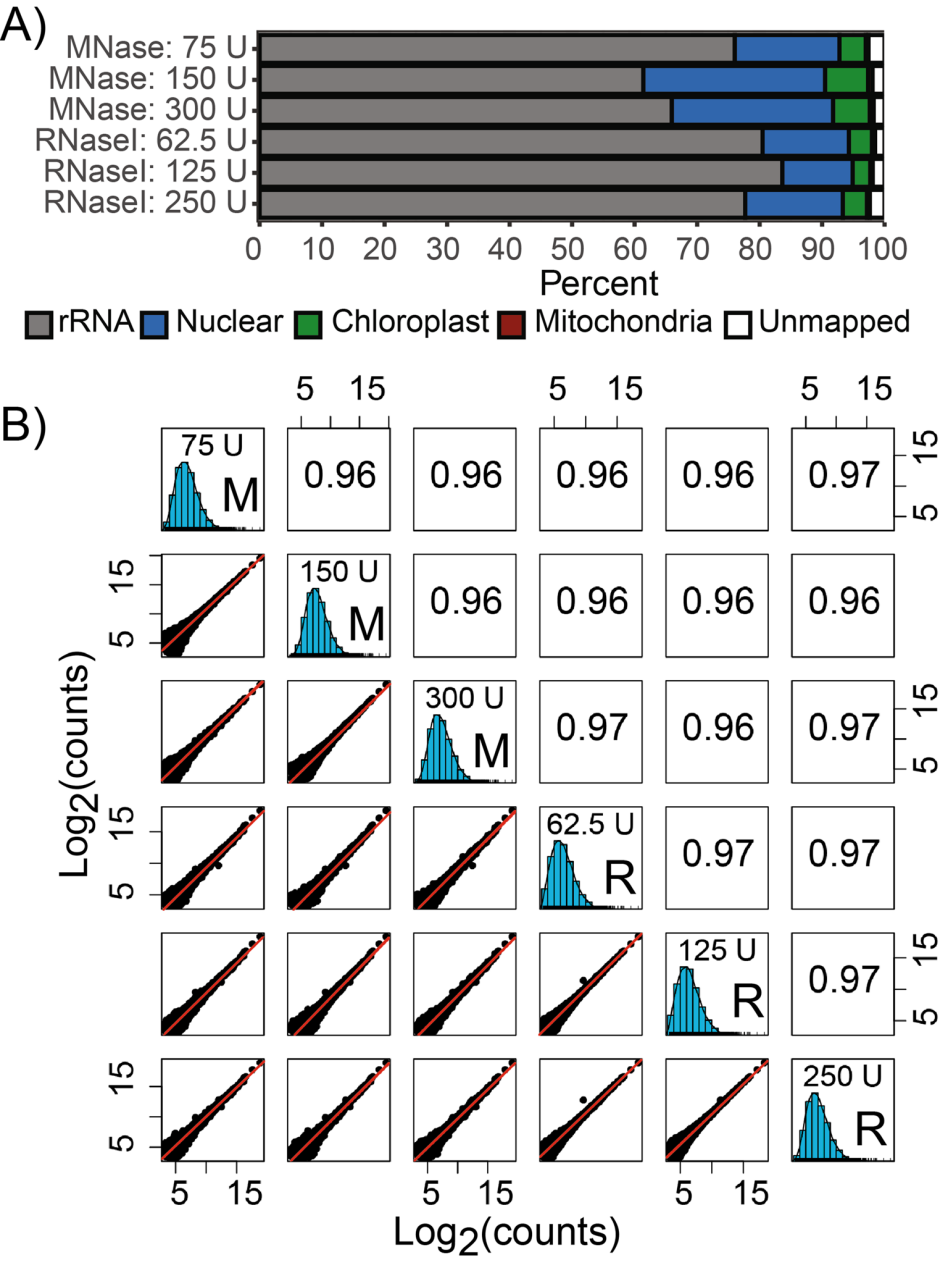
Quantitative comparison of Ribo-seq datasets generated over diverse nuclease treatments. **(A)** Ribo-seq library composition, based on RPF mapping location. **(B)** Spearman’s correlation of RPF density over annotated protein-coding sequences (CDS). Lowly translated genes with fewer than 10 counts were excluded from the analysis. The line of best fit is shown as the red diagonal. M, MNase-treated; R, RNase I-treated; U, Units of applied ribonuclease (note that the specified RNase activity was applied per 1 mL of plant lysate, derived from 100 mg of plant fresh weight tissue)

To expand on the polysome profile observations, 6 of the nuclease treatments were selected for Ribo-seq. As expected, rRNA dominates the library composition (Fig. 2A). Importantly, a crosswise comparison of RPF density over annotated genes displayed high correlations across all datasets (Fig. 2B), indicating that translatome data generated using MNase and RNase I are quantitatively comparable over a wide range of digestion conditions. These observations support the fair comparison of published datasets generated using different nuclease regimes, which is particularly relevant when attempting to integrate plant translatome data from chloroplast-focused studies that use MNase, to nuclear-focused studies that use RNase I.

**Fig. 3:**
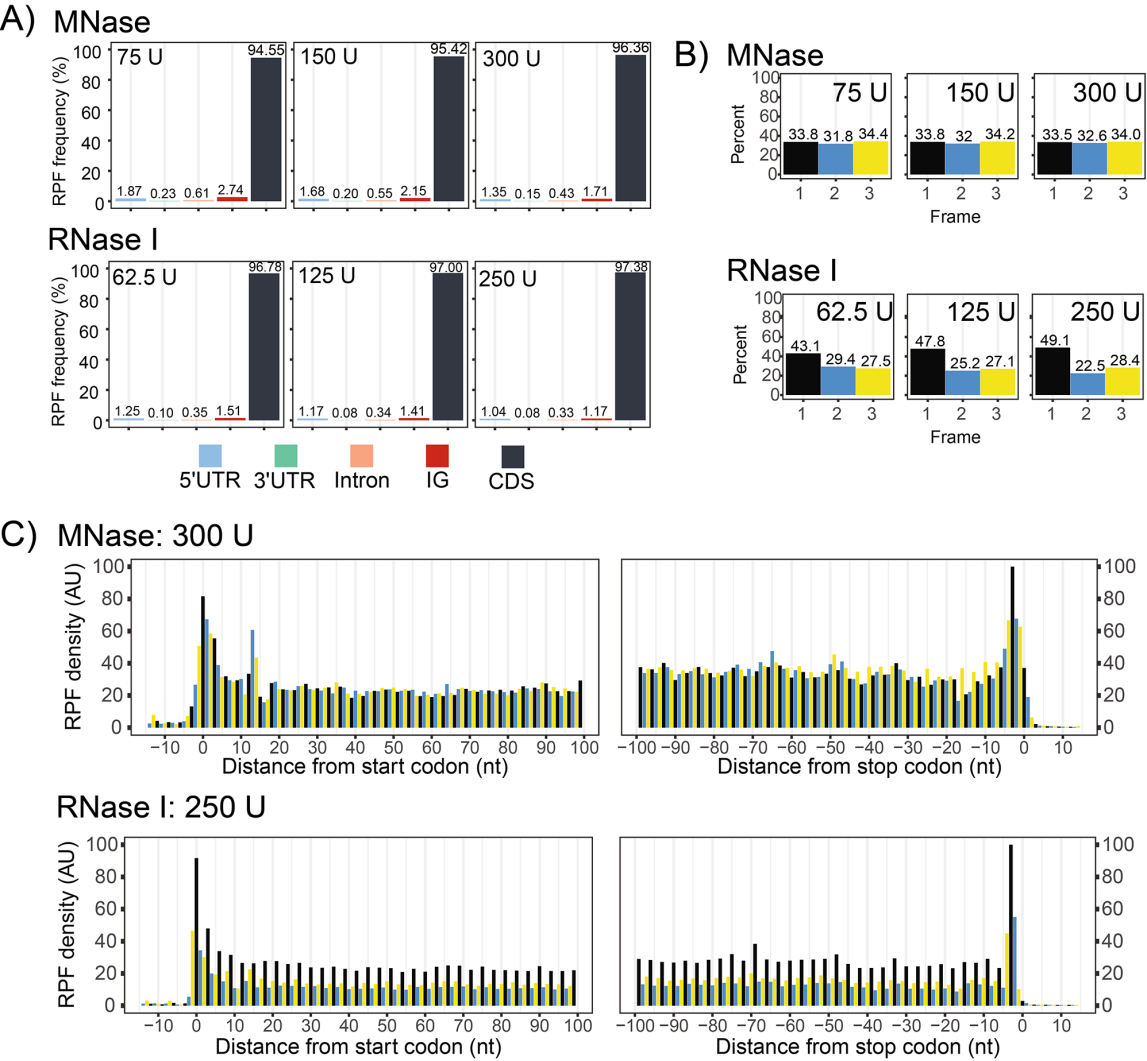
Qualitative comparison of Ribo-seq datasets generated over diverse nuclease treatments. **(A)** Proportion of mapped RPFs across genomic features. The percentages are calculated after removal of rRNA. **(B)** Quantification of RPFs in each frame of translation. Frame 1 is in reference to the annotated start and stop codons. **(C)** Metagene analysis for samples generated following digestion with 300 U of MNase, or 250 U of RNase I (note that the specified RNase activity was applied per 1 mL of plant lysate, derived from 100 mg of plant fresh weight tissue). RPFs are positioned at their P-site using offsets calculated from the 5’-position. U, Units of applied ribonuclease; UTR, untranslated regions; IG, intergenic regions; CDS, annotated protein-coding sequences

Next, the qualitative properties of each translatome were assessed. True RPFs should predominantly be found in annotated protein-coding sequences (CDS), which was indeed the case for all treatments (Fig. 3A). This was also confirmed through manual inspection of RPF density over selected genes (Figure S1). Since RPF density is reflective of translational kinetics, increased RPF density should be visible every 3 nucleotides as ribosomes slow down to decode each codon [22]. This pattern is commonly referred to as triplet periodicity and is often utilized as a quality measure and for the statistical detection of actively translated reading frames [3, 9, 40, 49, 50]. To measure triplet periodicity, RPFs were positioned at their P-site and the RPF density was quantified over the three frames of translation. Periodicity was only observed for samples treated with RNase I (Fig. 3B, C), reaffirming the previously described qualitative benefits of using RNase I over MNase [18].

**Fig. 4:**
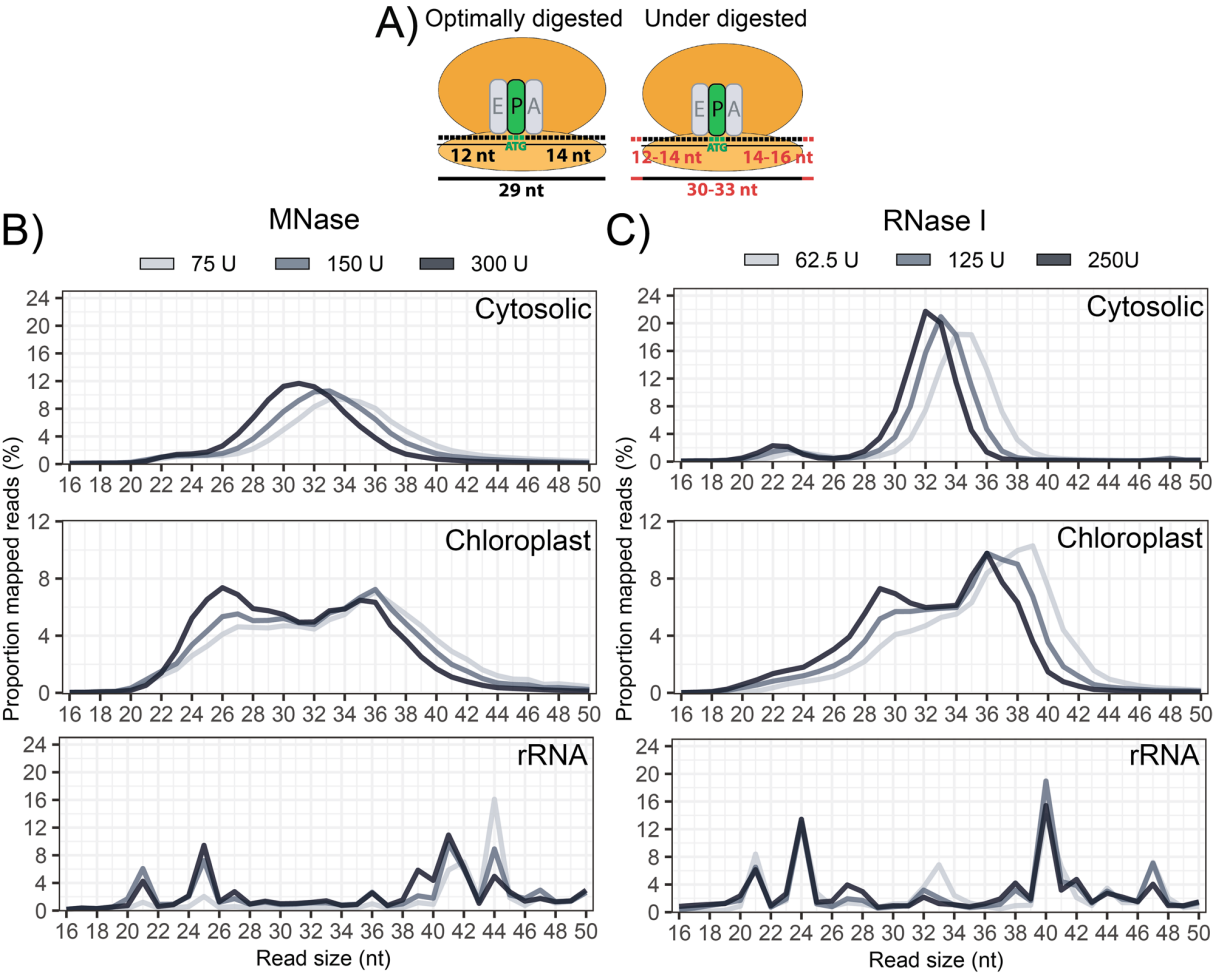
RPF size distribution across variable digestion conditions. **(A)** Schematic comparison between optimally digested and under-digested RPFs. Ribosomes decode the codon positioned in the ribosome P-site, which is typically 12 nt from the 5’ end from an optimally digested RPF. Exact P-site position is difficult to assess for under-digested RPFs, because of variable nucleotide digestion on the terminal ends (red). **(B-C)** Size distribution of RPFs mapping to the nuclear genome, the chloroplast genome, and rRNA, following digestion using **(B)** MNase or **(C)** RNase I

In plants, most cytosolic RPFs are reported to be ~ 28–29 nt in size which are characterized by strong triplet periodicity [8, 21]. RPFs larger than 28–29 nt tend to display lower triplet periodicity, which may be attributed to excess nucleotide(s) at either the 5’ or 3’ end (Fig. 4A) thereby confounding P-site estimations. Under the conditions tested, the majority of cytosolic RPFs in our samples were larger than 29 nt, indicating under digestion. However, the expected shift towards smaller sizes was observed as nuclease concentration was increased (Fig. 4B, C). This size shift was not observed for the rRNA, indicating robust protection of specific rRNA fragments. In addition, a secondary cytosolic RPF peak at ~ 20–24 nt was present in the samples, being more prominent with RNase I treatment (Fig. 4C). This secondary peak likely corresponds to ribosomes with an empty A-site [47], illustrating that diverse species of RPFs are captured, and highlighting the importance of using a broader size selection. Two peaks were also observed for chloroplast RPFs, which is a pattern also described in maize [6]. Additional analysis of these two populations are provided below in a specific section concerning chloroplast-derived RPFs.

**Fig. 5:**
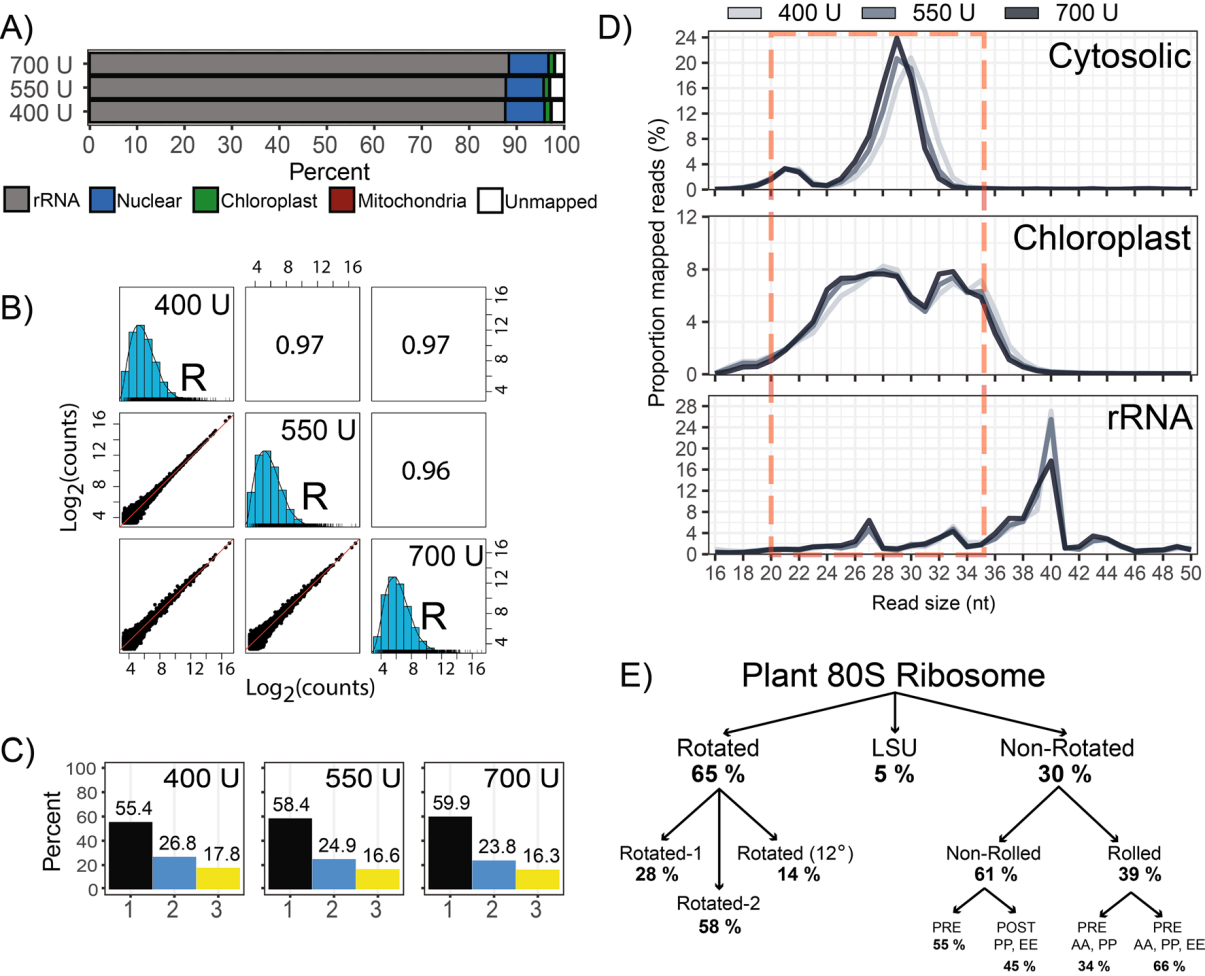
Refinement of RNase I treatment. Three additional Ribo-seq libraries were generated with increasing increments of RNase I treatment. **(A)** Ribo-seq library composition, as described in Fig. 2. **(B)** Spearman’s correlation of RPF density over annotated CDS (log_2_-counts) as described in Fig. 2. **(C)** Quantification of RPFs in the three frames of translation. Frame 1 is in reference to annotated start codons. **(D)** Size distribution of RPFs mapping to the nuclear genome, the chloroplast genome, and rRNA. The red dashed box indicates our new preferred RPF size selection to avoid major rRNA while including cytosolic and chloroplast RPFs. **(E)** Structural conformations of actively translating 80S cytosolic ribosomes from tobacco. The nomenclature and values were derived from CryoEM data from [45]. LSU, Large Subunit of the ribosome. U, Units of applied ribonuclease; R, RNase I

Given that the size of cytosolic RPFs were larger than expected, additional increasing increments of RNase I (400 U, 550 U, and 700 U) were tested to find the minimum concentration that efficiently produces cytosolic RPFs sized 28–29 nt. These Ribo-seq libraries were remarkable similar in composition, counts over gene CDS, and triplet periodicity (Fig. 5A-C), indicating that this digestion range very robustly generates reproducible data. Notable improvements in triplet periodicity were observed (55.4–59.9% RPFs in frame 1, Fig. 5C) compared to the under digested samples (43.1–49.1% RPFs in frame 1, Fig. 3B). When using 550–700 U of RNase I, the majority of cytosolic RPFs were stably around 29 nt (Fig. 5D). Of note, an independent study following a similar ribosome profiling methodology used 1400 U of RNase I, and reported similar periodicity values and RPF size distribution around 29 nt [8]. This suggests that digestion beyond 700 U (at least up to 1400 U) does not provide cost effective benefits. Together, these observations prompted us to apply 600 U RNase I as standard procedure.

Actively translating 80S ribosomes undergo numerous conformational changes during an elongation cycle. In tobacco, a recent Cryo-EM study [45] has revealed that in a given snapshot, the majority of cytosolic ribosomes are found in the rotated (65%) and non-rotated (~ 30%) states (Fig. 5E). Although our triplet periodicity values are relatively low compared to other studies, we note that they stabilize at around 60%, which is similar to the proportion of ribosomes found in the rotated conformation. Since our protocol was optimized to minimize nuclease treatment, we speculate that the stabilized periodicity values around 60% are reflective of the diverse ribosome conformations. Although it is undeniable that higher periodicity can provide greater confidence in classifying actively translated ORFs, our experience with non-periodic datasets (generated using MNase) is that the observation of RPF density near the putative start and stop codon of an ORF is more than sufficient. In addition, the RPF distribution between non-periodic and tri-periodic datasets is highly similar, with the majority of reads mapping to CDS (Fig. 3A). ORF detection programs often require a minimum number of reads along an ORF, which argues that increasing sequencing depth provides more benefits than either selecting only triperiodic reads or improving tri-periodicity (potentially introducing bias in the native distribution of different ribosome conformations; Fig. 5E). For these reasons, we focused our efforts into rRNA removal, which is the most cost-effective way to increase the number of informative reads (i.e., RPF coverage).

### Removal of contaminating rRNA fragments

**Fig. 6:**
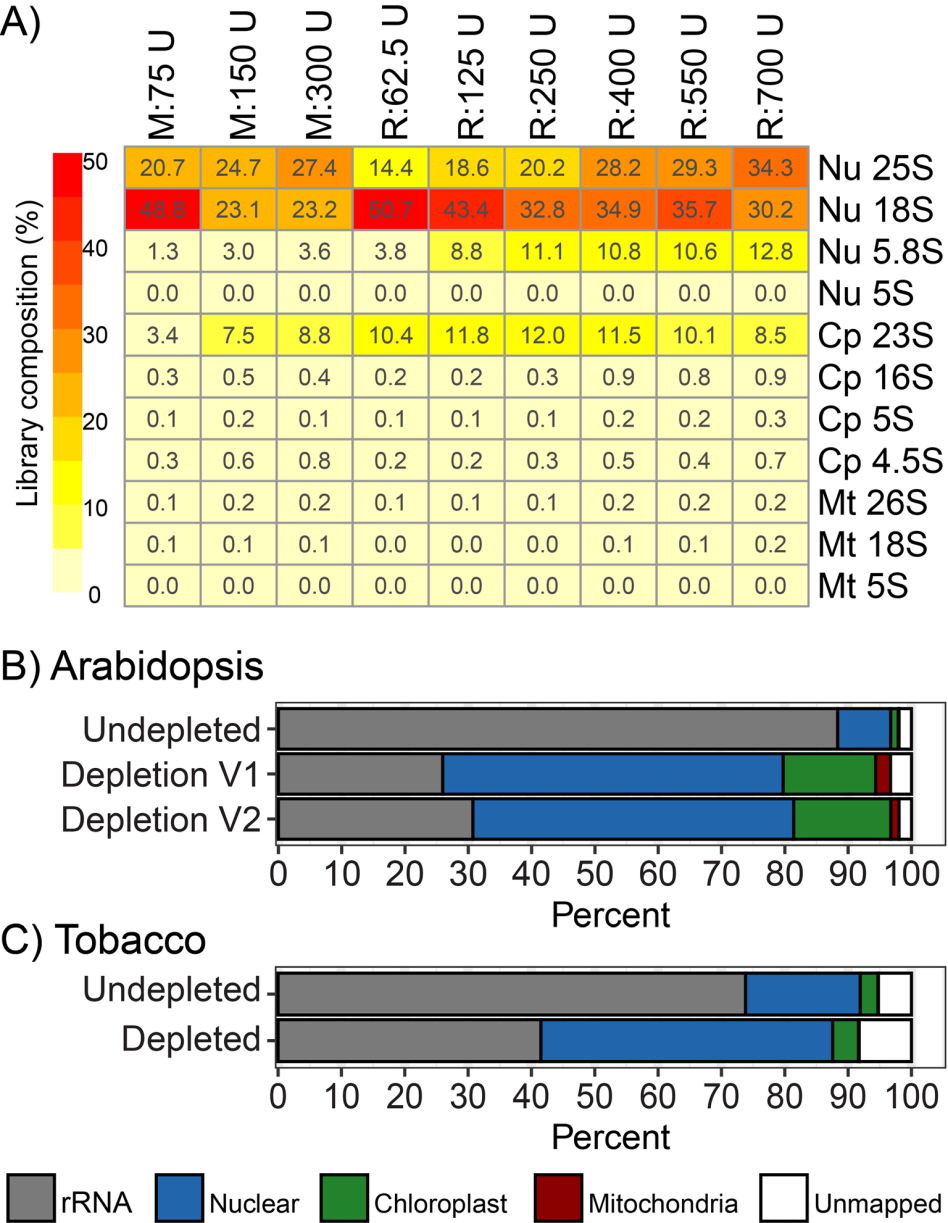
Removal of contaminating rRNA fragments from Ribo-seq datasets. **(A)** Contribution of individual rRNA species in Arabidopsis Ribo-seq libraries generated following different ribonuclease treatments. **(B)** Arabidopsis Ribo-seq library composition following rRNA depletion with depletion cocktail version 1 (V1) containing 24 oligos and depletion cocktail version 2 (V2) containing 29 oligos. The undepleted library corresponds to the 700 U RNase I-treated sample described above (Fig. 5A). **(C)** Tobacco Ribo-seq library composition following rRNA depletion. The sequences of the biotinylated oligos used for rRNA depletion are provided in Supplemental Tables S1 and S2. Nu, nuclear-encoded; Cp, chloroplast-encoded; Mt, mitochondria-encoded; U, Units of applied ribonuclease; M, MNase-treated; R, RNase I-treated

Our initial Ribo-seq datasets were generated by selecting RPFs from 20 to 50 nt, to ensure that the majority of chloroplast RPFs were captured. We now recommend a size selection of 20–35 nt, which still captures the majority of chloroplast RPFs, while simultaneously excluding the very abundant rRNA fragments at ~ 40 nt (Fig. 5D). When broken down, the most problematic rRNA fragments belong to the nuclear-encoded 25 S, 18 S, and 5.8 S rRNAs (Nu 25 S, Nu 18 S, Nu 5.8 S) and the chloroplast-encoded 23 S rRNA (Cp 23 S), irrespective of nuclease treatment or plant material (Fig. 6A). The sum of all other rRNA species contributed less than 2.5%, and therefore their contaminating effect is neglectable. Next, we identified high coverage rRNA regions that were repeatedly detected across several datasets, and designed biotinylated oligos to target these regions for removal (Fig S2). While designing the oligos, we noticed that the relative abundance of some rRNA fragments displayed high variation, even among technically similar replicates. For example, two fragments derived from the Nu 18 S and the Cp 23 S differed by 15% and 10% of the total library size, respectively, from two libraries that differed only by PCR (Figure S3B). Since PCR can have such a profound effect on the abundance of rRNA fragments, we reasoned that rRNA removal is most robust when performed prior to PCR amplification (ideally prior to any enzymatic step) because the molar ratios of oligos to the contaminants are best maintained. We thus formulated our initial Arabidopsis depletion cocktail (Version 1, Table S1) targeting the top 24 most abundant rRNA fragments and performed rRNA depletion directly on the gel-purified RPFs. Following this procedure, we effectively reduced rRNA contamination from 85% to ~ 25%, which corresponds to a 7-fold improvement in informative reads (Fig. 6B). Examination of the rRNA-depleted dataset revealed that new rRNA fragments began to disproportionately dominate the library, prompting us to add five additional oligos to our depletion cocktail (Version 2, Table S1). Surprisingly, the extra oligos did not yield any benefits (Fig. 6B), indicating that there is a limitation in the number of oligos that will result in noticeable improvements. Indeed, oligo cocktails containing 60 oligos report only a 50% rRNA reduction [8] which is less efficient than our 29 oligos. When designing depletion oligos for new plant species, we recommend ranking the contaminating rRNA fragments by abundance, and report consistent depletion results when targeting the top 29 most abundant fragments. However, 2–5 rRNA fragments can account for more than 90% of a Ribo-seq library [2], so a minimal cocktail containing only 5–10 oligos may already provide sufficient benefits for most applications.

### Preservation of rRNA fragments and their spatial distribution within the 80S ribosome

**Fig. 7:**
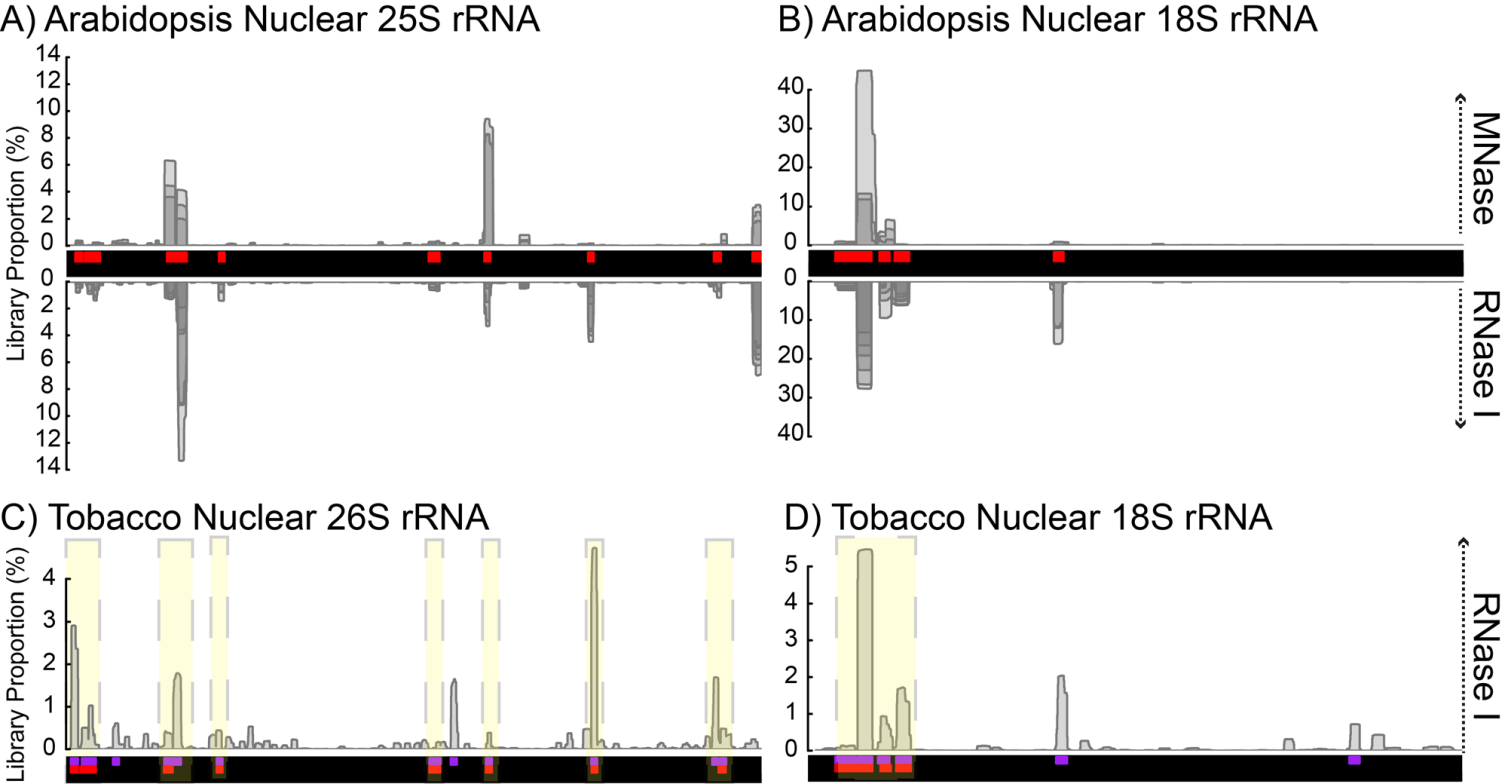
Preservation of contaminating 80S rRNA fragments. **(A-B)** Contaminating rRNA fragments under different ribonuclease regimes for the Arabidopsis nuclear-encoded **(A)** 25 S and **(B)** 18 S rRNAs. The coverage is derived from all Ribo-seq libraries described above that were generated with MNase (above y-axis) and RNase I (below y-axis). Regions with overlapping coverage from multiple libraries are shown in darker shades of grey. Arabidopsis depletion oligos (red) are given in Table S1. Similar plots for all rRNA species are shown in Figure S2. **(C-D)** Contaminating rRNA fragments across plant species, illustrated within the tobacco nuclear-encoded 26 S **(C)** and 18 S rRNAs **(D)**. Depletion oligos used for tobacco (purple) are described in Table S2. For comparative purposes, the Arabidopsis depletion oligos (red) were aligned to tobacco rRNA genes, and are displayed on equivalent positions. Yellow shades indicate preserved rRNA contaminants between Arabidopsis and tobacco. Similar plots for all rRNA species are available in Fig S4.

As mentioned at the beginning, an initial concern was that increasing nuclease digestion would create more rRNA fragments. However, our data demonstrates that this is not the case over a wide range of nuclease concentrations. Despite the observation that RNase I concentrations higher than 250 U caused monosome breakdown (Fig. 1C), we did not observe altered rRNA distributions (Fig. 5D) or higher rRNA contamination (Figs. 2A and 5A) from treatments with higher nuclease concentrations, suggesting that no new fragments are formed. In fact, a closer examination revealed that the most abundant rRNA fragments are preserved across all our datasets, irrespective of the plant material or ribonuclease treatment (Fig. 7A, B and Fig S2). Furthermore, many of the rRNA fragments identified in Arabidopsis are also present in tobacco Ribo-seq datasets (Fig. 7C, D and Fig S4), suggesting that similar fragments are also preserved across plant species (i.e., in rRNAs orthologs).

**Fig. 8:**
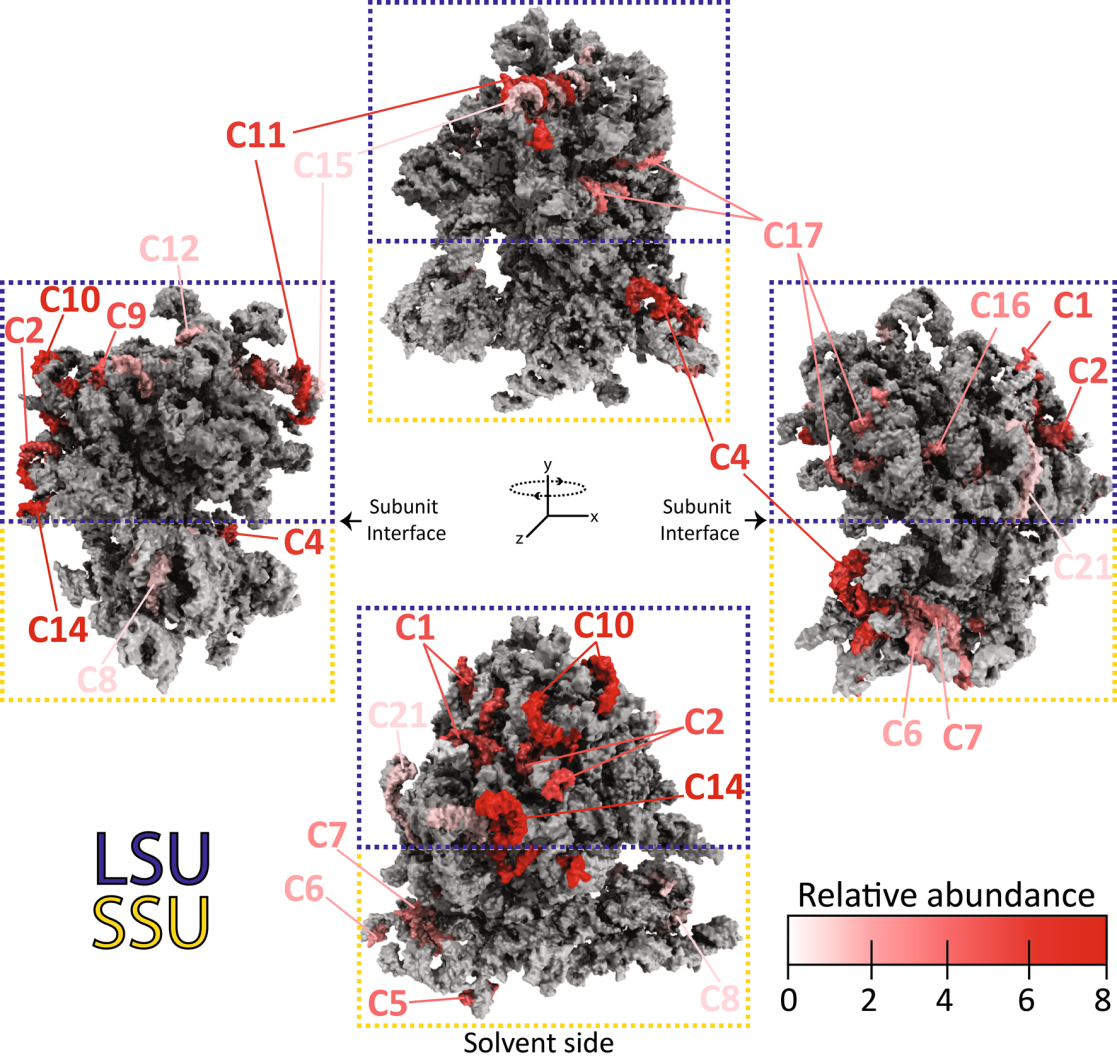
Mapping of contaminating rRNA fragments to the tobacco 80S ribosome structure. Major contaminating rRNA fragments (C1-C21) identified from tobacco Ribo-Seq datasets (Table S2) were mapped onto the molecular model of the tobacco 80S ribosome (PDB: 8B2L). The four rRNA molecules are shown in gray as spheres. Contaminants are colored in shades of red that reflect their relative abundance within our Ribo-seq datasets (additional details provided in Table S2). The ribosomal proteins are excluded from the model for clarity. The large subunit (LSU) and small subunit (SSU) are indicated in the blue and yellow dashed boxes, respectively

Together, these observations prompted us to explore the spatial distribution of the most abundant rRNA fragments within the plant 80S ribosome to gain insights into their origin. To this end, we used the recently solved cryo-EM structure of the tobacco 80S ribosome (PDB: 8B2L)(Smirnova et al., [45]. The analysis confirmed that many of the rRNA fragments that regularly contaminate Ribo-seq datasets are derived from surface exposed rRNA helices which are not shielded by ribosomal proteins (Fig. 8). The RNase nick sites occur more frequently on rRNA hairpins, loops, and bulges. Several of the most abundant contaminants (C1, C2, C10 and C14) are located on the solvent-exposed surface of the ribosome, which is where rRNA expansion segments (ESs) are predominantly localized [53]. In contrast, few contaminants were localized at the subunit interface (contact site of the small and large subunits). The interface contains the three tRNA-binding sites (A, P, and E), the decoding center, and the peptidyl transferase center [53], and is well shielded from the environment. We reason that fragments corresponding to the interface and other well-protected regions of the ribosome are likely to be larger than 50 nt, and are thereby excluded following our applied RPF size selection (20–50 nt).

These results highlight that many of the commercial rRNA depletion kits used for RNA-seq cannot perform well in Ribo-seq experiments. This is especially true for kits that contain a limited number of probes that target highly conserved rRNA sequences. Such probes are unlikely to correspond to the same fragments generated following nuclease treatment, and are not combined in optimal molar ratios. It is also worth noting that we have attempted using our Arabidopsis depletion oligos on tobacco samples, which was anticipated to be effective given the fragment similarities. However, only moderate depletion was achieved, which could be attributed to tobacco-specific single nucleotide polymorphisms (SNPs) that presumably hindered hybridization of the Arabidopsis oligos. Thus, a universal plant Ribo-seq depletion cocktail is unlikely to provide highly efficient rRNA removal across many plant species. Overall, these observations confirm the intuitive notion that major rRNA contaminants that dominate Ribo-seq datasets are formed from rRNA fragments whose 5’ and 3’ boundaries are readily accessible for ribonuclease attack. The most vulnerable regions belong to those located on the solvent-exposed surface of the ribosome. For the establishment of Ribo-seq in new plant species, these observations may facilitate the in silico prediction of major rRNA contaminants without any pioneer sequencing runs.

### Minimizing PCR bias

Ribo-seq protocols include a PCR amplification step, which can be a major source of bias when preparing sequencing libraries [11]. Indeed, we have observed that variation in PCR amplification can outweigh even differences in nuclease treatment (Figure S3A). To maximize reproducibility, libraries should be amplified to a similar concentration range using the same number of PCR cycles. In addition, libraries should not be amplified past the exponential phase of PCR, where the substrates of the PCR reaction become limiting and chimeric species begin to form. Although these notions seem trivial, we initially struggled with fulfilling both requirements because of highly variable PCR amplification across samples (12–19 cycles), despite using the same amount of RPF template. We suspect that this was caused by salts and/or pH altering molecules (or other contaminants) that co-purify with RPFs, and that negatively affect the enzymatic steps of the library preparation kit. This issue was alleviated by subjecting RPFs through an RNA purification column (e.g., NEB Monarch RNA cleanup) prior to library preparation, which has become a standard in our lab when using any library preparation kit. To ensure that libraries are amplified within the exponential phase of PCR, a qPCR approach was adopted to quantify the template prior to library amplification (see Supplemental Methods) as it has previously been described for Ribo-seq in non-plant species [34].

**Fig. 9:**
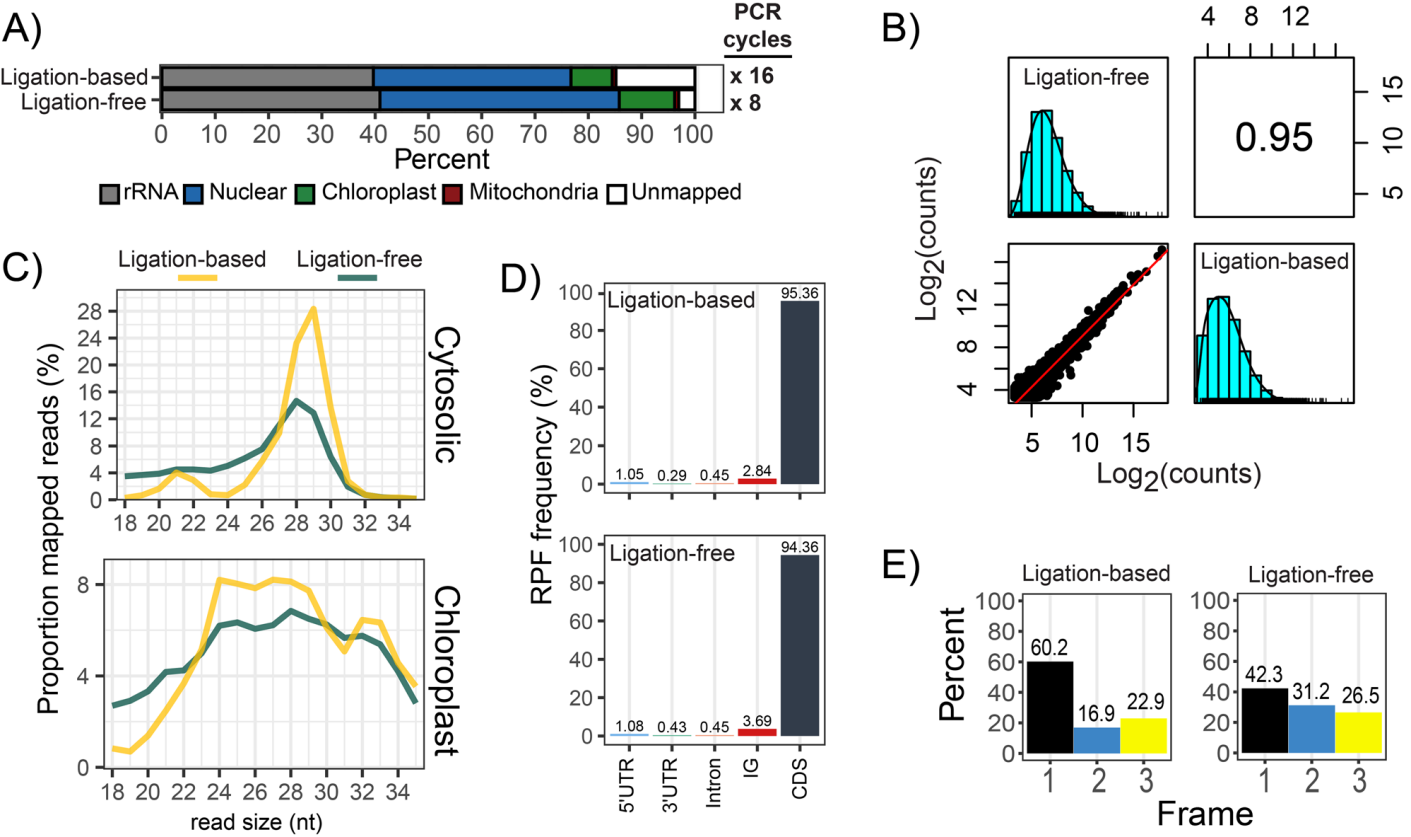
Comparison of ligation-based and ligation-free Ribo-seq strategies. Ligation-based strategies (e.g., NextFlex Small RNA-seq kit V4) use RNA-ligases for adapter attachment. Ligation-free strategies (e.g., Diagenode D-Plex small RNA-seq kit) use polyadenylation and reverse-transcription template switching for adapter incorporation. **(A)** Ribo-seq library composition. Indicated on the right are the number of PCR cycles required to obtain sufficient library quantities for sequencing. **(B)** Spearman’s correlation of RPF density over annotated CDS (log_2_-counts) as described in Fig. 2. **(C)** Size distribution of cytosolic RPFs mapping to the nuclear genome, and chloroplast RPFs mapping to the chloroplast genome. **(D)** Proportion of mapped RPFs across genomic features. The percentages are calculated after removal of rRNA. **(E)** Quantification of RPFs in the three frames of translation. Frame 1 is in reference to annotated start codons

Thus far, all of our Ribo-seq datasets were prepared using RNA-ligase based strategies, which display only moderate PCR efficiency when using rRNA-depleted samples as input. An appealing alternative are ligation-free approaches which utilize the template-switching ability of selected reverse transcriptases. These strategies are tailored for samples with low RNA input, and have already been successfully applied for Ribo-seq in mammals [20]. To compare these two approaches in plants, we generated Ribo-seq data using a ligation-based kit (NextFlex small RNA-seq V4) and a ligation-free kit (Diagenode D-Plex small RNA-seq). The ligation-free approach was magnitudes more efficient, requiring only 8 PCR cycles to obtain sufficient library quantities for sequencing, compared to the 16 PCR cycles for the ligation-based approach (Fig. 9A). Expectedly, triplet periodicity was lower for the ligation-free approach, which is due to the inability to distinguish 3’-terminal adenosine nucleotides that were enzymatically added, from those 3’-terminal adenosine nucleotides that truly belong to RPFs. Despite the reduced periodicity, the quantitative translatomes were still highly comparable (Fig. 9B). Thus, for general applications where codon-resolution is not required (to detect, e.g., rare ribosome frame-shifting events), we recommend the ligation-free approaches which are more efficient and convenient.

### Analysis of Chloroplast RPFs

**Fig. 10:**
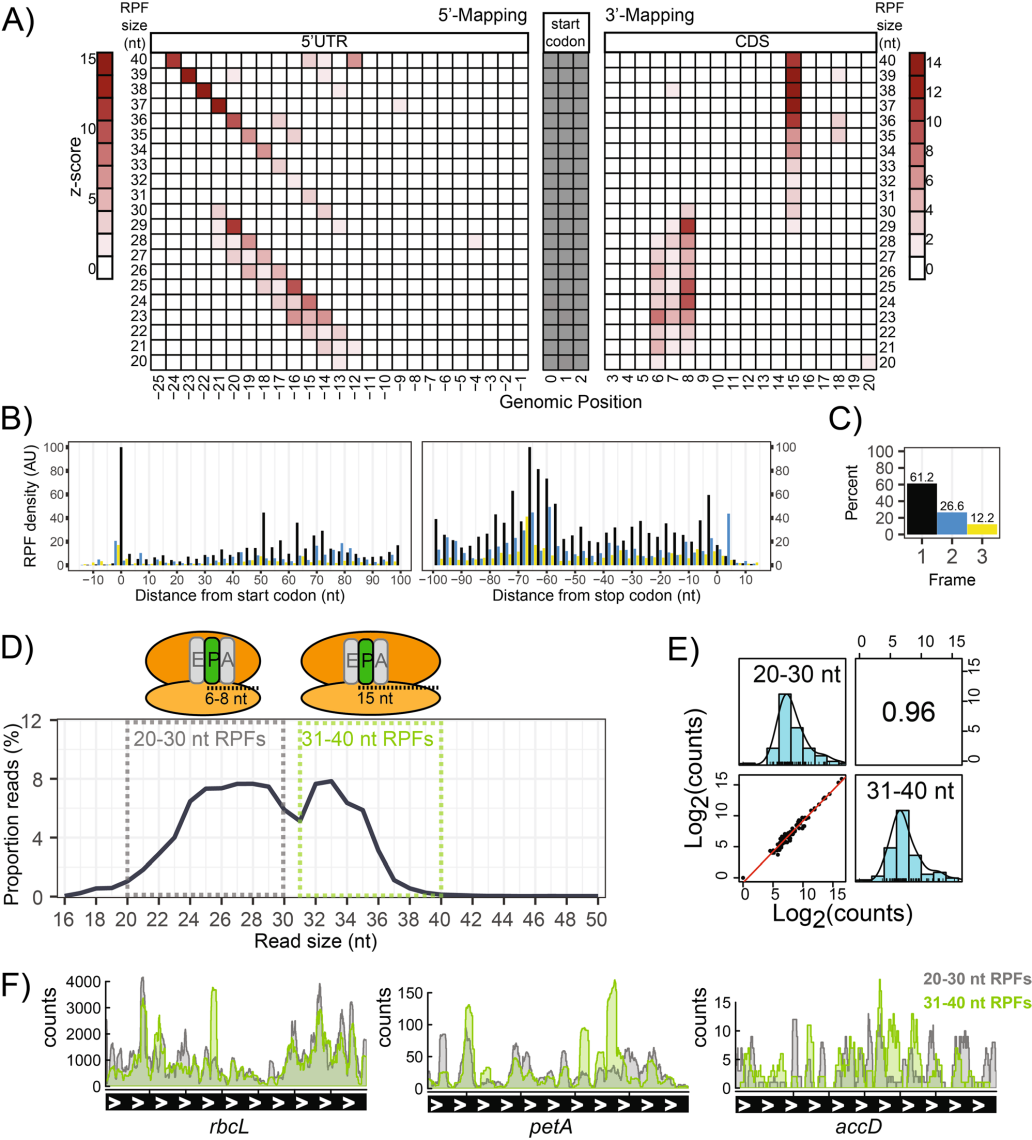
Properties of chloroplast RPFs. **(A)** P-site offsets. The RPF density around the start codon is normalized for each RPF size. Densities for 5’-mapped (left) and 3’-mapped (right) are both shown. **(B)** Metagene analysis for RPFs over the 79 annotated CDS genes of the chloroplast. RPFs are positioned at their P-site using the 5’-mapping offsets determined in **(A)**. **(C)** Quantification of RPFs in each frame of translation. Frame 1 is in reference to the start codon. **(D)** Bimodal RPF size distribution corresponds to unique P-site offsets. The small (20–30 nt) and large (31–40 nt) RPFs are indicated in dashed grey and green boxes, respectively. **(E)** Spearman’s correlation of RPF density over chloroplast CDS (log_2_-counts), for the small- and large-sized RPFs. **(F)** Coverage of the small- and large-sized RPFs over chloroplast genes. Highly expressed (*rbcL*), medium expressed (*petA*), and lowly expressed (*accD*) genes were selected to provide a broad representation. The data is derived from the library corresponding to the 700 U RNase I treatment (described in Fig. 5)

Plants harbor three translationally active compartments: the cytosol, mitochondria and plastids (predominantly chloroplasts in green tissue). While cytosolic RPFs clearly dominate plant Ribo-seq libraries and mitochondrial RPFs are neglectable, chloroplast RPFs make up a substantial fraction (Fig. 6). Due to the fact that essential proteins of the photosynthesis machinery are chloroplast-encoded, chloroplast translation is essential to establish photosynthesis. For studies that focus solely on chloroplast translation, we find that library sizes of 2–5 Million reads (after rRNA depletion) provide sufficient coverage for the vast majority of chloroplast genes. Due to the structural differences between the eukaryotic 80S ribosome of the cytosol, and the prokaryotic-like 70 S ribosome of the plastid, it is recommended that P-site offsets are estimated separately for these two ribosome species. The P-site offsets for cytosolic RPFs are predominantly 12–13 nt from the 5’end (Figure S5), which is the norm for eukaryotic RPFs [26]. In contrast, the P-site offsets for the chloroplast RPFs are diverse, and decrease from the 5’end as the RPF gets smaller (Fig. 10A). This indicates preferential nuclease digestion from the 5’end, which is a pattern that has previously been observed for chloroplast RPFs [6]. It was reported before that the determination of chloroplast P-site offsets can be performed by applying a constant 7 nt from the 3’end [6]. When applying 3’mapping to our own dataset, similar offset values (6–8 nt) were only observed for smaller RPFs (20–30 nt), whereas the larger RPFs (31–40 nt) displayed a constant 15 nt offset (Fig. 10A). It should be noted that chloroplast metagene analyses are inherently noisier since most land plant chloroplast genomes only encode ~ 80 CDS genes. Furthermore, some chloroplast transcripts are polycistronic with very short spacers in between reading frames (or even overlapping reading frames), thereby making it difficult (or impossible) to distinguish terminating ribosomes from initiating ribosomes around these short spacers. Despite these limitations, triplet periodicity is still visible across chloroplast genes (Fig. 10B, C).

Interestingly, the small and large RPFs that are characterized with the distinct P-site offsets, correspond to the two visible peaks in the RPF size distribution (Fig. 10D). To explore this further, chloroplast RPFs were size separated in silico, to determine if the small and large RPFs display unique localization patterns. Both RPF populations were similarly distributed across all chloroplast genes indicating no bias towards specific genes (Fig. 10E). For eukaryotic ribosomes, smaller sized RPFs (~ 19–21 nt) have been reported as stalled ribosomes containing an empty A-site [47]. This is unlikely to be the case for the small RPFs of the chloroplast, since they are relatively abundant (Fig. 10D) and are widespread along the entire CDS (Fig. 10F). Hence the molecular cause for the two observed RPF sizes remains to be determined. It is tempting to speculate that the small and large RPF populations of the chloroplast represent different rotational conformation states of actively translating ribosomes. For comparison, we also performed an in silico analysis of the small (18–24 nt) and large (25–34 nt) cytosolic RPFs, which also displayed moderate correlation across annotated CDS (Figure S6B). Since the small RPFs are much less abundant, it is difficult to compare the raw coverage. However, upon normalization, we did notice a tendency for small RPFs to be more abundant near start codons (Figure S6C-F).

### Complementary transcriptome

For calculating translation efficiency (TE), complementary RNA-seq libraries are typically generated in parallel with Ribo-seq libraries. Since the Ribo-seq dataset described here were generated from optimization trials, complementary transcriptomes were not generated, so no TE calculations are provided. However, we want to share our experiences with transcriptome generation: The depletion of rRNA from total RNA is standard in RNA-seq, with the most popular methods being enrichment of polyadenylated (poly(A)) transcripts, subtractive hybridization with biotinylated oligos, and enzymatic digestion. Since chloroplast RPFs contribute substantially to the plant translatome, we prefer strategies that preserve chloroplast transcripts, which is why we avoid poly(A) mRNA enrichment (since chloroplast transcripts are regularly not polyadenylated). We have also tested commercial rRNA removal kits that utilize subtractive hybridization, and have had good experience from oligos derived from riboPOOLs (siTOOLs Biotech). However, we observed a new problem that arises following efficient rRNA removal: New abundant RNA species begin to disproportionately dominate the RNA-seq library. For this reason, we currently prefer to use enzymatic based depletion strategies (e.g., Zymo-Seq RiboFree Total RNA Library Kit) which remove abundant RNA species in a sequence-independent manner. This strategy removes most rRNA, preserves organelle transcripts, and prevents any single RNA species from becoming disproportionately over represented.

## Conclusions

The genome-wide analysis of translation was revolutionized by ribosome profiling, which is often optimized across different labs to suite individual purposes. Through our own optimization efforts for Arabidopsis and tobacco, the methodology described here focuses on minimizing nuclease treatment and preserving chloroplast RPFs. This necessitates a broader RPF size selection, which comes at the expense of lower triplet periodicity. However, it has been demonstrated that non-periodic data (generated from MNase) still provides accurate translational dynamics [16, 17, 43, 46, 55]. Therefore, we instead prioritize sequencing depth, which we believe to be the limiting factor when trying to identify lowly translated ORFs. For this reason, we emphasize rRNA removal, which we find to be very efficient when performed at the RNA level, prior to any enzymatic steps. For a typical Ribo-seq experiment, we aim for ~ 20 million CDS mapped reads per sample. Thus, we typically sequence 40–60 million reads, depending on the efficiency of rRNA depletion. In addition, our structural assessment of rRNA fragments provide new insights that should benefit the general community when establishing ribosome profiling in new plant species. Together with our ribosome profiling protocol for the green alga *Chlamydomonas reinhardtii* [19], this provides a tool box that paves the way for highly comparative Ribo-seq studies in a wide range of plant species.

## Electronic supplementary material

Below is the link to the electronic supplementary material.

Supplementary Material 1

Supplementary Material 2

Supplementary Material 3

Supplementary Material 4

Supplementary Material 5

Supplementary Material 6

Supplementary Material 7

## Data Availability

The sequencing data have been deposited in NCBI’s Gene Expression Omnibus under accession number GSE226508.
