# Supplementary figures and images for: Optimization of ribosome profiling in plants including structural analysis of rRNA fragments
Source: Plant Methods. 2024 Sep 16;20:143. doi: 10.1186/s13007-024-01267-3 (PMC11406806; doi:10.1186/s13007-024-01267-3)

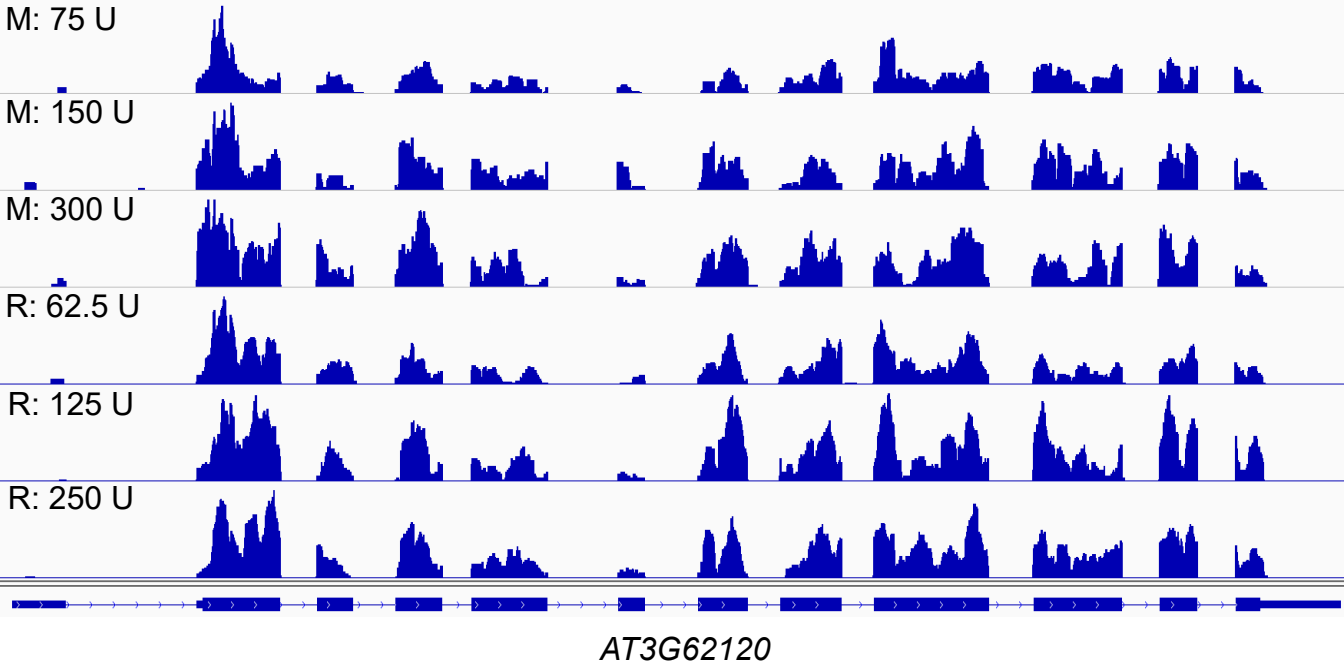

Supplement: Supplementary file 1 — Supplementary Material 1 [file 13007_2024_1267_MOESM1_ESM.pdf]

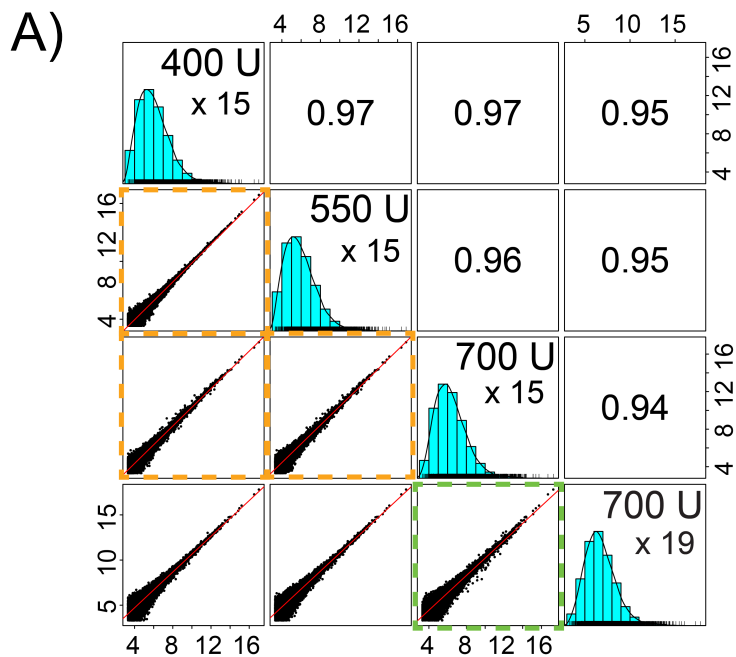

**B)**

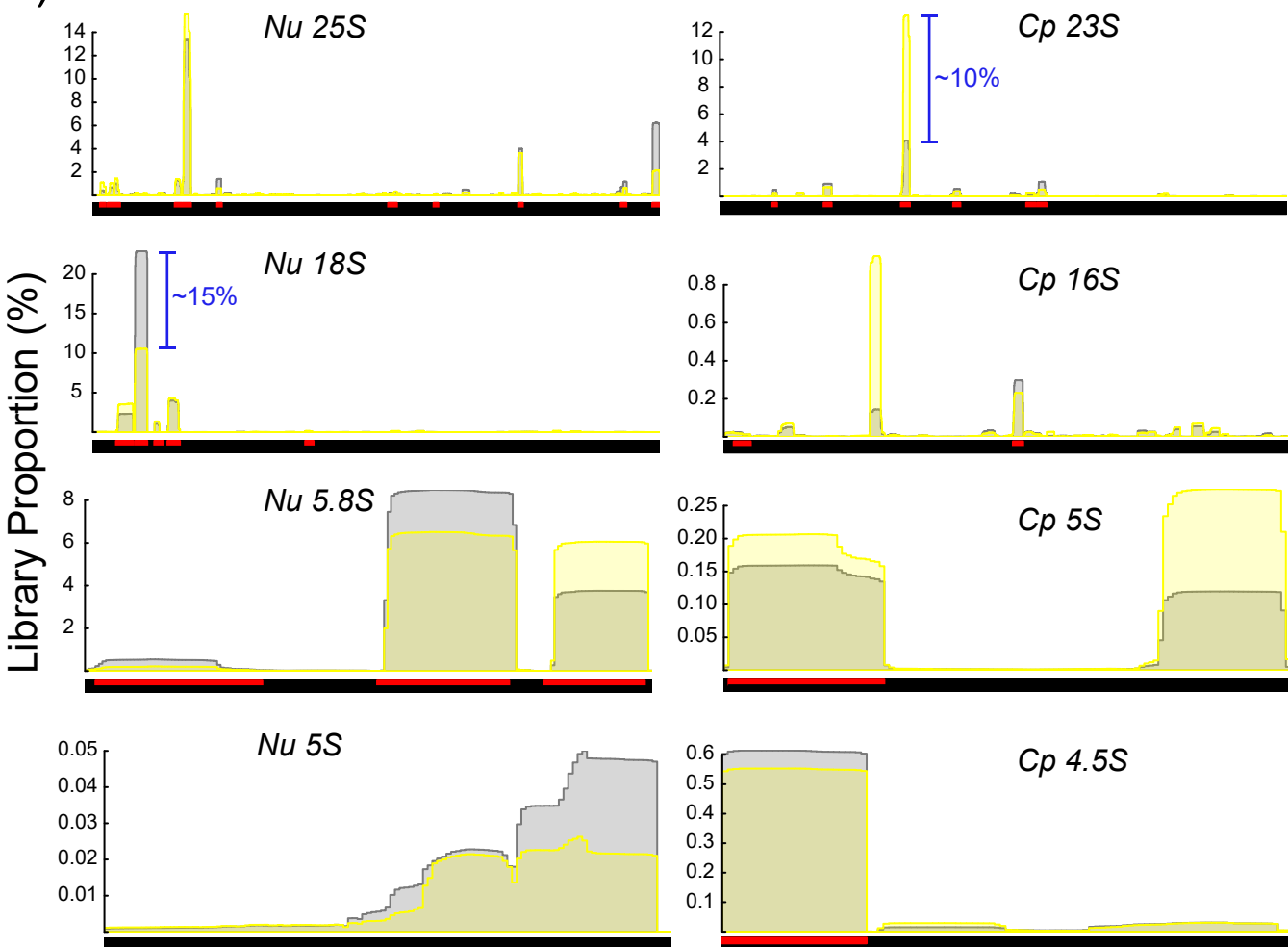

Supplement: Supplementary file 3 — Supplementary Material 3 [file 13007_2024_1267_MOESM3_ESM.pdf]

Library Proportion (%)

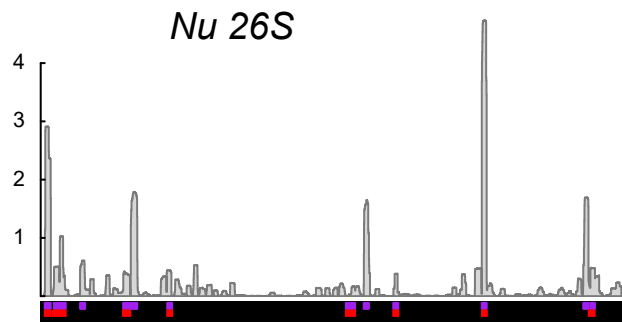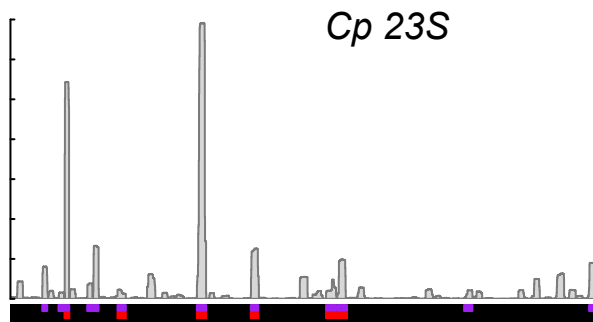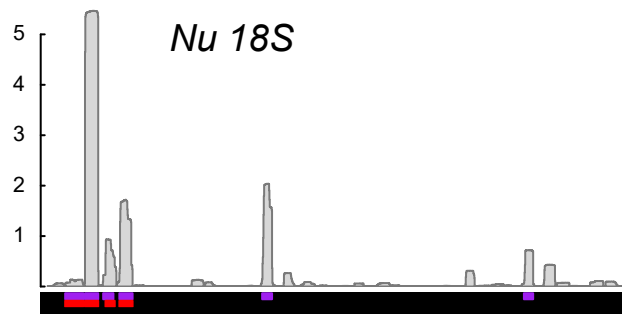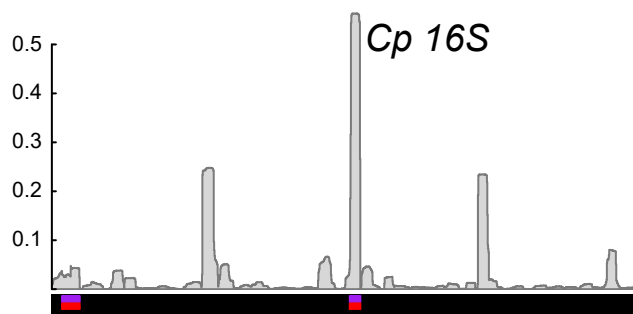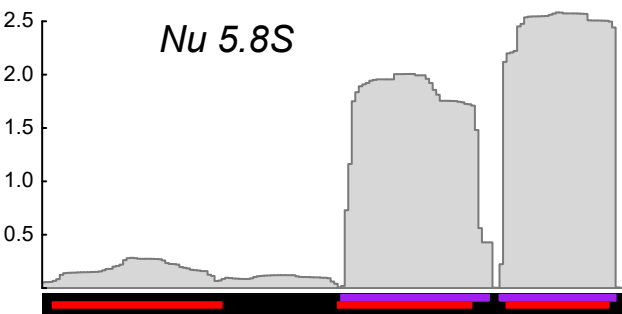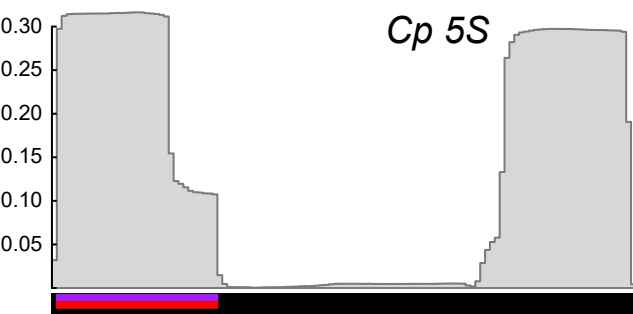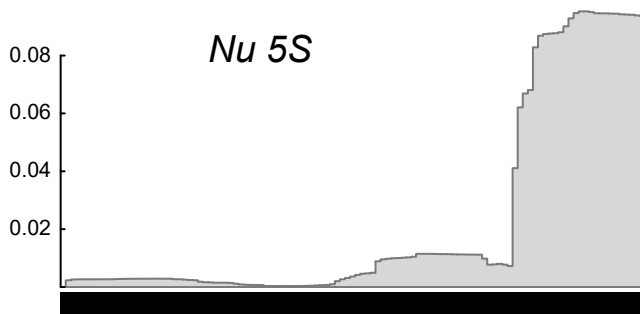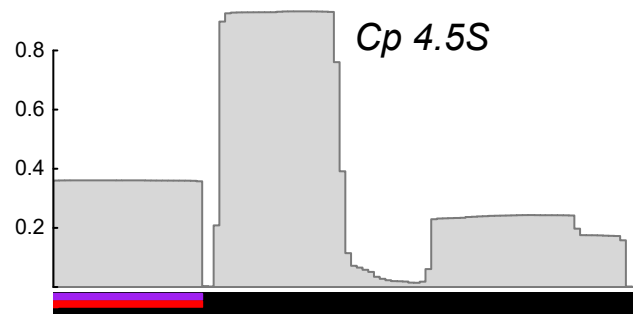

Supplement: Supplementary file 4 — Supplementary Material 4 [file 13007_2024_1267_MOESM4_ESM.pdf]

# Cytosolic RPFs: Start codon

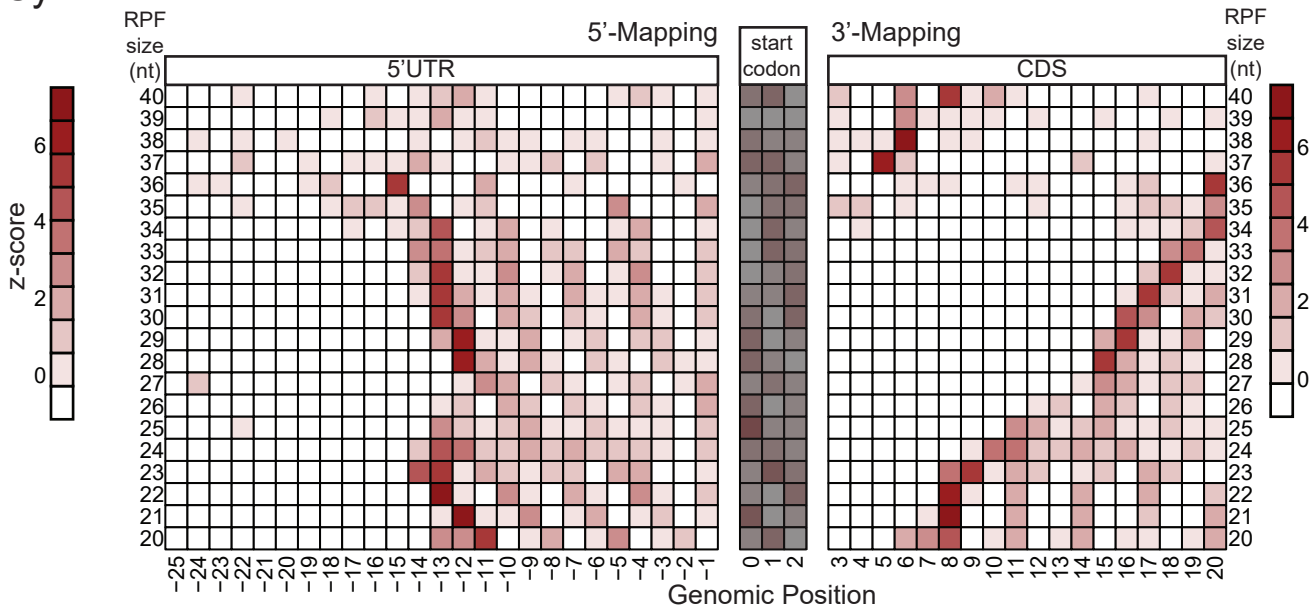

Supplement: Supplementary file 5 — Supplementary Material 5 [file 13007_2024_1267_MOESM5_ESM.pdf]

A)

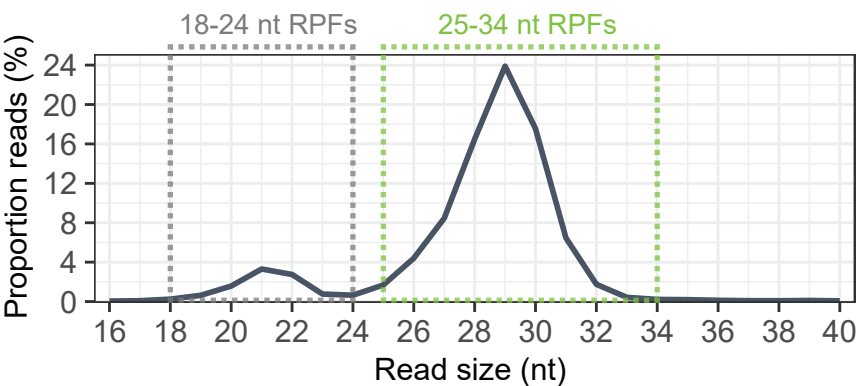

B)

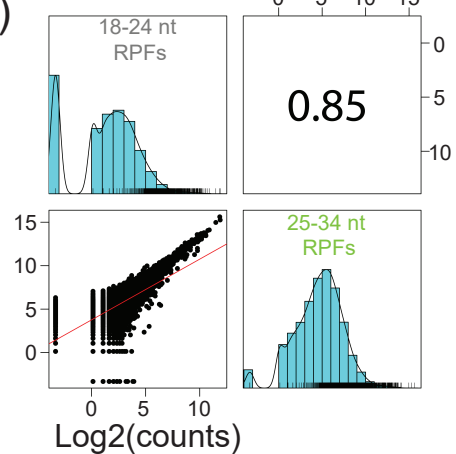C) *AT2G39730 (RCA)*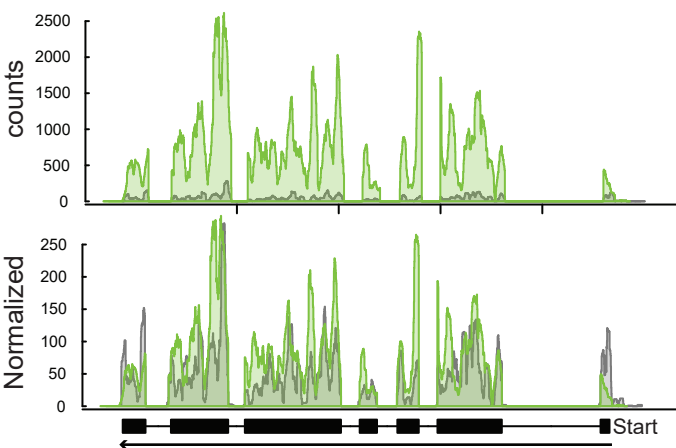D) *AT1G67090 (RBCS-1A)*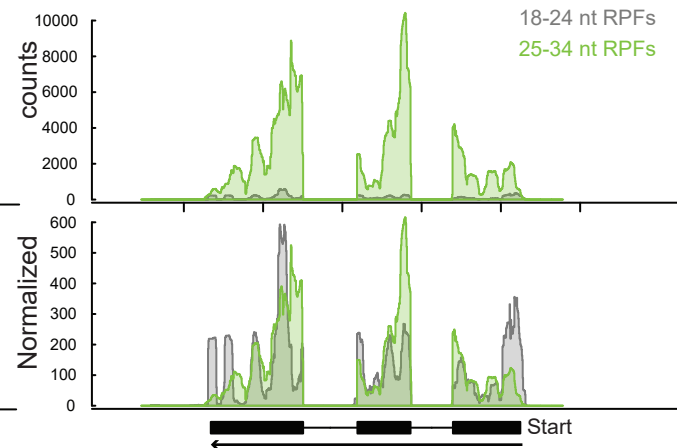E) *AT1G29930 (LHCB1.3)*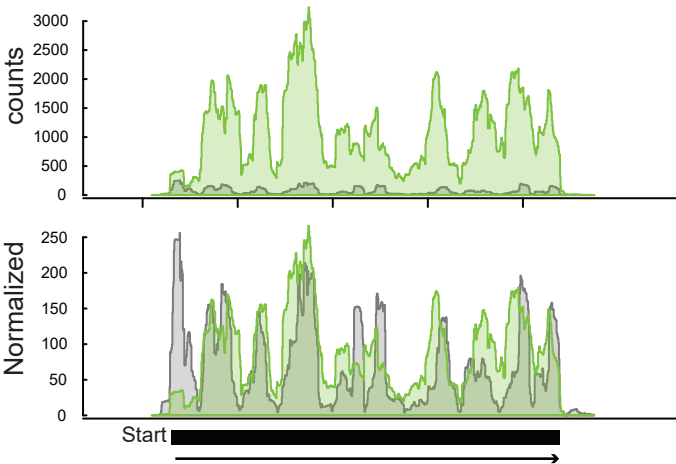F) *AT1G79040 (PSBR)*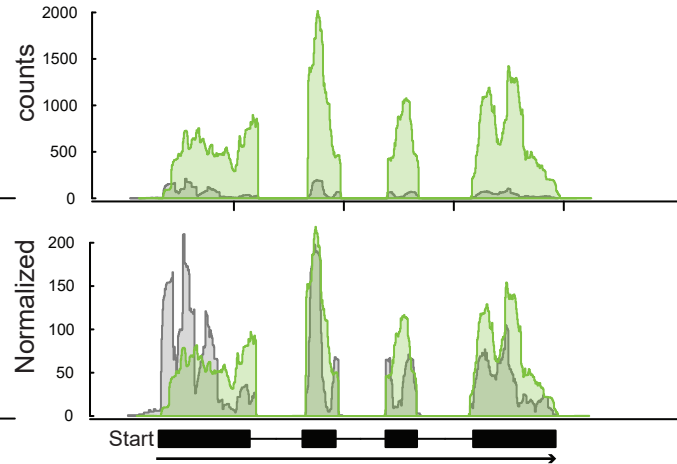

Supplement: Supplementary file 6 — Supplementary Material 6 [file 13007_2024_1267_MOESM6_ESM.pdf]
