# Supplementary material for: Optimization of ribosome profiling in plants including structural analysis of rRNA fragments
Source: Plant Methods. 2024 Sep 16;20:143. doi: 10.1186/s13007-024-01267-3 (PMC11406806; doi:10.1186/s13007-024-01267-3)
Supplement: Supplementary file 2 — Supplementary Material 2 [file 13007_2024_1267_MOESM2_ESM.pdf]

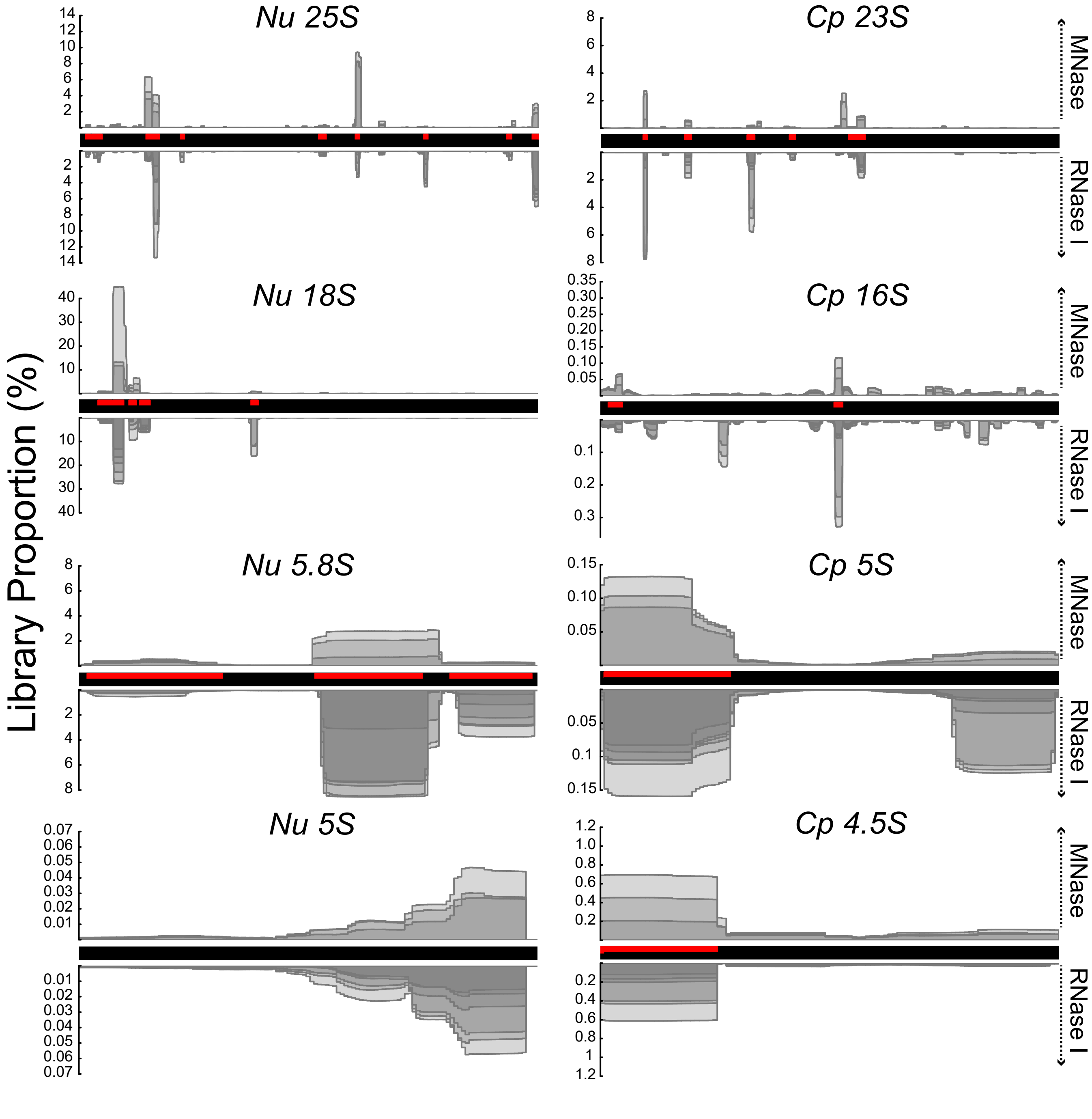

Nu 25S
Cp 23S
Nu 18S
Cp 16S
Nu 5.8S
Cp 5S
Nu 5S
Cp 4.5S
MNase
RNase I
Library Proportion (%)
