# Supplementary material for: Optimization of ribosome profiling in plants including structural analysis of rRNA fragments
Source: Plant Methods. 2024 Sep 16;20:143. doi: 10.1186/s13007-024-01267-3 (PMC11406806; doi:10.1186/s13007-024-01267-3)
Supplement: Supplementary file 7 — Supplementary Material 7 [file 13007_2024_1267_MOESM7_ESM.docx]

# Supplemental Data

**Figure S1.** RPF coverage is maintained in annotated CDS across tested digestion conditions. The coverage profile emphasizes that different digestion conditions do not perturb RPF mapping, and that isolated RPFs are indeed derived from CDS regions. Shown is the RPF coverage over *LHCA2* (AT3G62120), which was selected on the basis of having high RPF coverage and multiple exons. For the gene structure, thick bars represent CDS, thin bars represent exons, and lines represent introns. M, MNase-treated; R, RNase I-treated; U, Units of applied ribonuclease.

**Figure S2.** Preservation of contaminating rRNA fragments in diverse Arabidopsis RPF libraries. Coverage of reads mapped along Arabidopsis nuclear-encoded and chloroplast-encoded rRNA species derived from all Arabidopsis Ribo-seq libraries described in this manuscript that were treated with MNase (above y-axis) and RNase I (below y-axis). The read coverage is normalized to the size of each library, with the relative abundances shown as a percentage of the total library size. Note the different y-axis scales which are due to variable contamination contributions of different rRNA species. Regions with overlapping coverage from multiple libraries are shown in darker shades of grey. Designed rRNA-depletion oligos are shown as red lines (23-55 nt long). The x-axis is scaled according to the full length of each rRNA species, and ranges from 102-3382 nt.

**Figure S3.** Effects of PCR amplification bias on Ribo-seq datasets. A replicate Ribo-seq library was generated using the same RPFs derived from our 700 U RNase I treatment (described in Figure 5). The PCR amplification efficiency of this replicate library was lower (amplified in 19 cycles) than the original library (amplified in 15 cycles), allowing for the assessment of PCR bias. **(A)** Correlation plot highlighting that differences in PCR reduces translatome comparability to a greater extent than differences in nuclease treatment. The number of PCR cycles used to amplify each library (x15 or x19) are shown. The libraries that only differ in nuclease treatment (orange boxes) are stronger correlated (R=0.96-0.97) than the libraries that only differ by PCR (green box, R=0.94). This is even true for libraries generated using different nuclease enzymes (Figure 1C, R=0.96-0.97). **(B)** Differences in PCR cycle number affect the relative abundance of specific rRNA contaminants. Shown is the read coverage along rRNA species, for the two technically similar libraries. The library amplified with 15 cycles is colored grey, whereas the library amplified with 19 cycles is colored yellow. The vertical blue bars highlight contaminating rRNA fragments with substantial differences in relative abundance. The red bars show the location of Arabidopsis rRNA depletion oligos (Table S1). Each rRNA species is scaled according to its full length. U, Units of applied ribonuclease; Nu, Nuclear-encoded rRNA; Cp, Chloroplast-encoded rRNA.

**Figure S4.** Preservation of contaminating rRNA fragments across plant species. Coverage of reads mapped along tobacco nuclear-encoded and chloroplast-encoded rRNA species derived from a tobacco Ribo-seq dataset (Details as shown in Figure S7). Tobacco rRNA depletion oligos are shown in purple (Table S2). Arabidopsis rRNA depletion oligos are shown in red (Table S1) and were mapped to tobacco rRNA sequences to highlight equivalent regions. Some of the Arabidopsis oligos do not match perfectly to the tobacco rRNA sequences.

**Figure S5.** P-site estimation for cytosolic RPFs. The RPF density around the start codon is normalized for each RPF size. Densities for 5’-mapped (left) and 3’-mapped (right) are both shown. The P-site offset values selected for each RPF size were derived from the position of highest RPF density based on 5’-mapping, which are typically 12-13 nt upstream of the start codon. The data is derived from the library corresponding to the 700 U RNase I treatment (described in Figure 5).

**Figure S6.** Characterization of different sized cytosolic RPFs. **(A)** RPF size distribution of the small (18-24 nt, grey) and large (25-34 nt, green) cytosolic RPFs. **(B)** Spearman’s correlation of RPF density over annotated CDS (log_2_-counts), for the small- and large-sized RPFs. Genes with 0 counts in both populations were filtered out. A pseudo count of 0.1 was added to the remaining genes. **(C-F)** Coverage of the small- and large-sized RPFs over selected nuclear-encoded genes. Shown are the RPF coverage for AT2G39730 (C), AT1G67090 (D), AT1G29930 (E), and AT1G79040 (F). Raw counts are displayed on top, whereas counts normalized to the small-RPFs are displayed on the bottom. The data is derived from the library corresponding to the 700 U RNase I treatment (described in Figure 5).

**File S1**. Example scripts

Table S1. Custom biotinylated DNA depletion-oligos for rRNA removal from Arabidopsis RPF samples. Depletion oligos were designed for Arabidopsis rRNA loci with high coverage across all Arabidopsis Ribo-seq libraries produced from our lab (for details see Results). The molar ratio reflects the relative abundance of these contaminants among all our Ribo-seq libraries. The final oligo cocktail (version 2) contains all the oligos in this table, mixed at the indicated molar ratios. The preliminary oligo cocktail (version 1) contained only the oligos without an Asterisk (*). Nu, nuclear-encoded; Cp, chloroplast-encoded.

| **Oligo name** | **Oligo sequence** | **Molar Ratio** |
| --- | --- | --- |
| **Nu_5.8S_1** | GGGCGCAACTTGCGTTCAAAGACTCGATGGTTCACGGG | 10 |
| **Nu_5.8S_2** | GTGACACCCAGGCAGACGTGCCCTCGGCC | 4 |
| **Nu_5.8S_3*** | TACGTTCTTCATCGATGCGAGAGCCGAGATATCCGTTGCCGAGAGTCG | 2 |
| **Nu_18S_1** | TACCATCAAACAAACTATAACTGATTTAATGAGCCATTCGCAGTTTCACAGTCTG | 2 |
| **Nu_18S_2** | TGCACGTATTAGCTCTAGAATTACTACGGTTATCCGAGTA | 16 |
| **Nu_18S_3** | CGTCGACCTTTTATCTAATAAATGCGTC | 4 |
| **Nu_18S_4** | GGCCATGCGATCCGTCGAGTTATCATGAATCATCAGAGCA | 10 |
| **Nu_18S_5** | TCGCCGACCGAAGGGACAAGCCGACCA | 4 |
| **Nu_25S_1** | GGGAATCCTTGTTAGTTTCTTTTCCTCCGCTTAT | 2 |
| **Nu_25S_2** | CGTCCGATTTTCAAGCTGGGCTCTTCCCGGTTCGCT | 2 |
| **Nu_25S_3** | GAGGACGCTTCTCCAGACTACAATTCGAACGCCG | 2 |
| **Nu_25S_4** | AGCCCGGGCTTAGGCCGCCACCGTAATCCGCGTCGGTCCACG | 4 |
| **Nu_25S_5** | AGGCGCGTGCTGCAGACCACGATCACGGCAGCGACGTCTCCACAAGCGTAT | 8 |
| **Nu_25S_6** | AGATCAAGGTCGGTCGGCGGTGCACCCG | 1 |
| **Nu_25S_7** | CCGTTCCCAGTCCGTCCCCCGGCCGGCAC | 4 |
| **Nu_25S_8** | AGCGAGCCTTGGGACCAAAAACAGGGGT | 4 |
| **Nu_25S_9** | GCTCCTACTGAGGGTCGGCAATCGGGCGGCGGGC | 1 |
| **Nu_25S_10** | GTCGAATCTTAGCGACAAAGGGCTGAATCTCAGTGGATCGTGGCAGC | 10 |
| **Nu_25S_11*** | CTCGGTCCTCCGGATTTTCAAGGGCCGCCGGGGGCGCACCGGACACCACGCGA | 2 |
| **Cp_4.5S_1** | TAAACGGCTCGTCTCGCCGTGACCTTC | 2 |
| **Cp_5S_1** | TGGTGTTGTTCCTCTACGCCTAGGACACCAGAA | 1 |
| **Cp_16S_1** | ACGCACAGCGCCTAGTATCCATCGTTT | 1 |
| **Cp_16S_2*** | TCCCGTCCGACTTGCATGTGTTAAGCATGCCGCCAGCGTTCATCC | 1 |
| **Cp_23S_1** | TGCTCTCCCACAACCCCGTTTTC | 2 |
| **Cp_23S_2** | GGCTCCTCCCACTGCTTGGGAGCTTACGGTTTCATGTTCT | 1 |
| **Cp_23S_3** | GAGGTCATATCTAGTATTCAGAGTTTGCCTCGATTTGGTACCGCT | 8 |
| **Cp_23S_4** | AGGTCGTTCGAGCTTTTCCTGGGAGTATAGCATGGGTTACTTCAGCG | 4 |
| **Cp_23S_6*** | ATCCCACAGCTTCGGCAGATCGCTTAGCCCCGTTCA | 2 |
| **Cp_23S_7*** | CGCCTGGTACTCGAACATTGGCTCGGGGCATTTTCTCTACCCCTTCTT | 2 |

Table S2. Custom biotinylated DNA depletion-oligos for rRNA removal from tobacco RPF samples. Depletion oligos were designed for tobacco rRNA loci with high coverage across tobacco Ribo-seq libraries produced from our lab (includes datasets not described in this study). The molar ratio reflects the relative abundance of these rRNA fragments across our Ribo-seq libraries. The “Labels” of the rRNA fragments depicted in Figure 7, are listed accordingly. The “Ribosome regions” are named accordingly to Armache et al, 2010. Oligos prefixed “NTa” are used only for tobacco rRNA depletion, whereas those without prefix are used for both Arabidopsis and tobacco rRNA depletion. Nu, nuclear-encoded; Cp, chloroplast-encoded; LSU, large subunit; SSU, small subunit.

| **Oligo name** | **Oligo sequence** | **Molar ratio** | **Label (as in Fig. 7)** | **Ribosome region** |
| --- | --- | --- | --- | --- |
| **NTa_Nu_5.8S_1** | GTTTCGGGCGCAATTTGCGTCCAAAGATTCGATGGTTCACGG | 5 | C1 | H7/H5 |
| **NTa_Nu_5.8S_2** | GCGTGACGCCCAGGAAGACGTGCCCTCGGCCTA | 5 | C2 | ES^L^3/ES^L^4 |
| **Nu_18S_1** | TACCATCAAACAAACTATAACTGATTTAATGAGCCATTCGCAGTTTCACAGTCTG | 0.5 | C3 | h6/h7 |
| **Nu_18S_2** | TGCACGTATTAGCTCTAGAATTACTACGGTTATCCGAGTA | 6 | C4 | h8 |
| **NTa_Nu_18S_1** | CCGGCAGAAGGGACGAGACGACCGGTGCAC | 4 | C5 | ES6^S^a |
| **NTa_Nu_18S_2** | GACCTTTTATCTAATAAATGCATCCCTTCCA | 2 | C6 | ES^S^3a/c |
| **NTa_Nu_18S_3** | GGCCGTGCGATCCGTCGAGTTATCATGAATCATCGCAGCA | 3 | C7 | ES^S^3b/h10 |
| **NTa_Nu_18S_4** | CGTTGAATACATCAGTGTAGCGCGCGTG | 1 | C8 | h30/h41 |
| **Nu_25S_1** | GGGAATCCTTGTTAGTTTCTTTTCCTCCGCTTAT | 5 | C9 | H11/H13 |
| **Nu_25S_3** | GAGGACGCTTCTCCAGACTACAATTCGAACGCCG | 7 | C10 | ES^L^5, H15 |
| **NTa_Nu_25S_1** | TACCACAATCAAGGAGACGGCGTTCCACGGGCGTATCG | 6 | C11 | ES^L^7b |
| **Nu_25S_6** | AGATCAAGGTCGGTCGGCGGTGCACCCG | 1.5 | C12 | ES^L^9 |
| **Nu_25S_7** | CCGTTCCCAGTCCGTCCCCCGGCCGGCAC | 0.5 | C13 | ES^L^27 |
| **NTa_Nu_25S_2** | GCAAGCCTTGGGTCCAAAAAGAGGGGCA | 8 | C14 | ES^L^31 |
| **NTa_Nu_25S_3** | GCCCGGGCTTTGGCCGCCCCCCCAATCCACGCTGGTCCAC | 1 | C15 | ES^L^7b |
| **NTa_Nu_25S_4** | CCTACATTGTTCCATCGACCAGAGGCTGTT | 3 | C16 | H60/H61 |
| **NTa_Nu_25S_5** | CATGCGTCGCTTCTAGCCCGGATTCTGACT | 3 | C17 | H97/H94 |
| **NTa_Nu_25S_6** | AGGCCCCTACTGCGGGTCGGCAAGCGG | 0.5 | C18 | ES39b |
| **NTa_Nu_25S_7** | GAGCCGCCCGATTCTAAGGCTGGGCTGTTCCCG | 0.5 | C19 | H13/H14/ES5 |
| **NTa_Nu_25S_8** | AAACAACCCGACTCGTAGACAGCGCCTCG | 0.5 | C20 | H21 |
| **Nu_25S_11** | CTCGGTCCTCCGGATTTTCAAGGGCCGCCGGGGGCGCACCGGACACCACGCGA | 1 | C21 | H56/H59 |
| **Cp_4.5S_1** | TAAACGGCTCGTCTCGCCGTGACCTTC | 1.5 | - | - |
| **Cp_5S_1** | TGGTGTTGTTCCTCTACGCCTAGGACACCAGAA | 0.5 | - | - |
| **Cp_16S_1** | ACGCACAGCGCCTAGTATCCATCGTTT | 0.5 | - | - |
| **Cp_16S_2** | TCCCGTCCGACTTGCATGTGTTAAGCATGCCGCCAGCGTTCATCC | 0.5 | - | - |
| **Cp_23S_1** | TGCTCTCCCACAACCCCGTTTTC | 6 | - | - |
| **Cp_23S_2** | GGCTCCTCCCACTGCTTGGGAGCTTACGGTTTCATGTTCT | 0.5 | - | - |
| **Cp_23S_3** | GAGGTCATATCTAGTATTCAGAGTTTGCCTCGATTTGGTACCGCT | 10 | - | - |
| **Cp_23S_4** | AGGTCGTTCGAGCTTTTCCTGGGAGTATAGCATGGGTTACTTCAGCG | 3 | - | - |

**Optimized Plant Ribosome Profiling protocol**

*Isolation of ribosome-protected fragments and total RNA*

300 mg frozen plant tissue was homogenized in liquid nitrogen with a mortar and pestle followed by the addition of 3 mL ribosome extraction buffer (0.2 M sucrose, 0.2 M KCl, 40 mM Tris-OAc pH 8.0, 10 mM MgCl_2_, 10 mM 2-Mercaptoethanol, 2% (v/v) polyoxyethylene (10) tridecyl ether, 1% (v/v) Triton X-100, 100 μg/mL chloramphenicol, 100 μg/mL cycloheximide). After brief mixing, a 0.5 mL aliquot of the lysate was flash frozen and stored at -80 °C for later total RNA extraction using TRIzol reagent (ThermoFisher cat# 15596026). This total RNA can be used for standard RNA-seq experiments. This step is especially important if the calculation of translation efficiencies is desired (i.e., the normalization of Ribo-seq data to RNA-seq data). The remaining lysate was filtered through glass wool, followed by centrifugation for 10 min at 15,000 x *g* at 4 °C to remove cell debris. 2.5 mL of the clarified lysate were incubated with 1500 U of RNase I (Ambion cat# AM2294) for 1 h at room temperature with gentle rotation to degrade mRNA regions that are not covered and protected by translating ribosomes. The ribonuclease-treated lysate contains mainly monosomes which were loaded onto a 2 mL sucrose cushion (30% (w/v) sucrose, 40 mM Tris-Acetate pH 8.0, 100 mM KCl, 15 mM MgCl_2_, 0.1 mg/mL chloramphenicol, 0.1 mg/mL cycloheximide, 0.2% β-MeEtOH) and centrifuged (OptimaTM L-80 XP Ultracentrifuge - Beckman Coulter, SW55 Ti rotor) for 1.5 h at 303,800 x *g* at 4 °C. The supernatant was then carefully aspirated and the pelleted monosomes resuspended in 0.5 mL footprint isolation buffer (10 mM Tris pH 8.0, 1 mM EDTA pH 8.0, 100 mM NaCl, 1% (w/v) SDS, 0.1 M EGTA pH 8.0). The RNA was immediately extracted from this pellet using 0.5 mL TRIzol reagent. Next RPFs were size-selected through electrophoresis on a 12% denaturing polyacrylamide gel (19:1, acrylamide:bisacrylamide) prepared in 1x TBE buffer (89 mM Tris, 89 mM Boric Acid, 2mM EDTA pH 8.0) containing 8 M urea. To this end, 25 μg RNA were resuspended in 40 μL of ribosome footprint loading buffer (90% (v/v) deionized formamide, 20 mM Tris-HCl pH 7.5, 20 mM EDTA pH 8.0, 0.04% (w/v) bromophenol blue, and 0.04% (w/v) xylene cyanol) and denatured for 10 min at 70 °C. The gel was run in 1x TBE buffer with a constant power of 30 W at constant temperature of 12 °C (achieved by a cooling unit). Co-migrating pre-stained RNA ladder (Biodynamics Laboratory cat# DM253) was used to visualize the regions of the gel to excise RPFs (20-35 nt). RNA was eluted from the excised gel piece in 4 mL TESS (10 mM Tris pH 8.0, 1 mM EDTA pH 8.0, 0.1 M NaCl, 0.2% (w/v) SDS) by overnight incubation at 4 °C with gentle rotation. Eluted RNA was isolated with 4 mL of phenol:chloroform:isoamyl alcohol (25:24:1), followed by overnight ethanol precipitation at -20 °C. To increase purity and further narrow the volume, the RNA pellet was resuspended in 0.1 M NaCl (500 μL) and subjected to a second round of phenol:chloroform:isoamyl alcohol (25:24:1) extraction, followed by two washes with chloroform:isoamylalcohol (24:1) and overnight ethanol precipitation at -20 °C. The received RPF pellet was washed twice with 75% ethanol and resuspended in 20 μL RNase-free water. Typical yields are 200-600 ng of RPFs per 300 mg plant fresh weight (depending on tissue source, e.g., developmental stage, mutant phenotype, etc.).

*rRNA depletion*

RNase free Biotinylated oligos were purchased from metabion international (Planegg, Germany). Oligo hybridization was performed following an adapted protocol from (Kraus et al., 2019). 150 ng of gel-purified RPFs were mixed with the following components in a PCR tube: 4 μL deionized formamide, 1 μL 20X SSC (3M NaCl, 0.3M Na-Citrate, pH 7.0 with HCl), 2 μL EDTA (5 mM, pH 8.0), 0.7 μL biotinylated oligo mix (100 µM), and RNase free water up to 20 μL. Hybridization of the oligos was performed in a thermocycler with heated lid using a slow temperature ramp according to the table below:

| Temp (°C) | 80 | 75 | 70 | 65 | 60 | 55 | 50 | 45 | 40 | 35 |
| --- | --- | --- | --- | --- | --- | --- | --- | --- | --- | --- |
| Time (min) | 5 | 2 | 2 | 2 | 2 | 2 | 2 | 2 | 2 | 2 |
| Ramp rate | -0.1°C/s | | | | | | | | | |

After oligo hybridization, each sample was topped off to 40 µL using a solution of 1X SCC and 20% formamide and kept at 35 °C until ready for oligo removal, which was performed using Dynabeads MyOne C1 (Thermo Fisher cat# 65002). For each sample, 45 µL of beads were washed according to manufacturer’s protocol for RNA applications, and divided into 30 µL and 15 µL aliquots for performing two rounds of oligo removal. Each sample was incubated with the 30 µL bead aliquot at room temperature for 15 min, and magnetized for 2 min (the supernatant contains the partially rRNA-depleted sample). The second round of oligo removal was performed by aspirating the supernatant into the 15 µL bead aliquot and repeating the incubation and magnetization. The final supernatant contains the rRNA-depleted RPFs, which was aspirated, transferred into a clean tube and ethanol precipitated overnight at ‑20 °C. The rRNA-depleted RPF pellet was resuspended in 42 µL RNase-free water and treated with TURBO DNase (Thermo cat# AM2238) in a 50 µL reaction according to the manufacturer’s instructions, in order to remove traces of leftover DNA oligos. Following DNase treatment, RPFs were purified using the Monarch RNA cleanup kit (NEB cat# T2030) following the modified protocol for small RNA, and eluted in 35 µL RNase-free water. Typical RPF yields following depletion are ~30% (i.e., 50 ng rRNA-depleted RPFs from 150 ng gel-purified undepleted RPFs).

*RPF Library preparation*

For ligation-free Ribo-seq, rRNA depleted RPFs were directly used as input into the D-plex small RNA-seq kit (Diagenode cat#C05030001) according to manufacturer’s instructions, and amplified with 7-9 PCR cycles. For RNA-ligase Ribo-seq, rRNA-depleted RPFs were treated with T4 polynucleotide kinase (PNK; ThermoFisher, cat#EK0031) in a two-step reaction, to prepare the terminal ends for adapter ligation. First, the depleted RPFs (~50 ng in 35 µL) were added to a 50 µL PNK reaction without ATP, for 10 min at 37 °C to promote the dephosphorylation reaction (removal of 3’ phosphate groups and 2', 3' cyclic monophosphate, which are typical end products of MNase and RNase I digestion). Next, ATP was added to the reaction, followed by another incubation for 30 min at 37 °C. The end-repaired RPFs were purified using the Monarch RNA cleanup kit using the modified protocol for small RNA, and eluted in 12 µL RNase-free water. These purified RPFs were immediately used as input for the NEXTflex small RNA-seq kit v3 (Perkin Elmer, cat# NOVA-5132-06) or v4 (Perkin Elmer, cat#NOVA-5132-31), according to the manufacturer’s instructions. The resulting cDNA was stored at -80 °C until the number of PCR cycles required for library amplification, was experimentally determined by qPCR (see below). In the final PCR amplification step of NEXTflex library preparation, the cycle number was adjusted according to the qPCR results, and typically require 14-16 PCR cycles.

*qPCR to determine the cycle number for Ribo-seq library PCR amplification*

From our experience, NEXTflex libraries that are <1 ng/µL are very difficult to multiplex in equimolar ratios, due to less accurate qubit quantification. In contrast, libraries >10 ng/µL can be considered to be overamplified and are outside the exponential PCR doubling range, creating technical biases in the results. The following qPCR protocol was designed to produce libraries within the range of 1.5-6.0 ng/µL. The primers are derived from the NEXTflex small RNA-seq kit v3:

| F primer | AATGATACGGCGACCACCGAGATCTACACGTTCAGAGTTCTACAGTCCGA |
| --- | --- |
| R primer | GTGACTGGAGTTCCTTGGCACCCGAGAATTCCA |

The instructions of the NEXTflex kit were followed up to STEP-F, eluting the cDNA product in 21 uL of elution buffer. Subsequently, 1 µL of this purified cDNA product was combined with 10 µL 2X Power SYBR Green PCR master mix (catalog# 4368577), 2 µL forward primer (0.5 µM), 2 µL reverse primer (0.5 µM), and 5 µL water (total volume of 20 µL). This qPCR reaction is aliquoted into 3 technical replicates (5 µL reactions per well), and run on an Applied biosystems 7900 HT machine with the following settings:

| 95 °C | 95 °C | 60 °C | 72 °C |  | 72 °C | Dissociation curve |
| --- | --- | --- | --- | --- | --- | --- |
| 2 min | 20 sec | 30 sec | 15 sec |  | 2min |  |
| x1 | x40 | | |  | x1 |  |

Based on the absolute Ct values obtained in this qPCR, the remaining 20 µL of the cDNA were PCR-amplified according to the table below:

| **qPCR Ct value** | **Recommended cycles** **in the final PCR amplification step of NEXTflex library preparation** | **Notes** |
| --- | --- | --- |
| **12** | **10** | Generally, the lower the number of PCR cycles used, the better the library quality (e.g., 12 cycles is better than 15 cycles). |
| **13** | **10** |  |
| **14** | **11** |  |
| **15** | **11** |  |
| **16** | **12** |  |
| **17** | **12** |  |
| **18** | **13** |  |
| **19** | **13** |  |
| **20** | **14** |  |
| **21** | **14** |  |
| **22** | **15** |  |
| **23** | **15** |  |
| **24** | **16** |  |
| **25** | **16** |  |
| **26** | **17** |  |
| **27** | **17** |  |
| **28** | **18** | Libraries in this range typically contain adapter dimers and display higher rates of PCR duplication. The translatome data can be used for the general analysis of differential translation, but it is not recommended for detecting rare translation events (e.g., upstream open reading frames) |
| **29** | **19** |  |
| **30** | **20** |  |
| **31+** | **N/A** | Libraries in this range will contain significant adapter-dimers. Thus, it is not recommended to sequence these libraries. |

Absolute Ct values may vary across machines and applied qPCR kits. Hence, it is recommended to calibrate these values to your machine and kits, according to the final library concentration in pioneer experiments.

*RNA seq library preparation*

Although we do not include RNA-seq libraries in this manuscript, this is important to include in many ribosome profiling experimental setups to calculate translation efficiencies (as RPF abundancies normalized to transcript abundancies). In green plant tissue, up to a quarter of the RPFs are of chloroplast origin, so we prefer to use RNA-seq strategies that also preserve chloroplast transcripts. We routinely use the Zymo-Seq RiboFree total RNA library kit (Zymo cat# R3000/R3003) whose protocol depends on an enzymatic degradation of rRNAs.

*Bioinformatics analysis*

*Arabidopsis thaliana:* The Arabidopsis GTF gene annotations (v45) and TAIR10 genome were downloaded from ensembl (https://plants.ensembl.org/index.html). Cutadapt (Martin, 2011) was used to remove adapters and UMIs. Ribo-seq contaminants are generally defined as rRNA, tRNA and snoRNA. These sequences were extracted from the gene annotation file, and converted to fasta format using bedtools (Quinlan and Hall, 2010). Importantly, the rRNA gene annotations from TAIR10 (AT2G01010, AT2G01020, AT3G41768, AT3G41979, ATMG00020, ATMG01380, ATMG01390, ATCG00920, ATCG00950, ATCG00960, ATCG00970, ATCG01160, ATCG01170, ATCG01180, and ATCG01210) do not include the nuclear 25S and 5S. Thus, these sequences were obtained from NCBI (25S rRNA; X52320.1) and the 5S rRNA database (5S; http://combio.pl/rrna/). Contaminants were filtered out using STAR aligner (Dobin et al., 2013) with the following parameters: *--outFilterMismatchNoverLmax 0.1 --outReadsUnmapped Fastx –outSAMmultNmax 1*. In the datasets described here, tRNA and snoRNA contribute negligible amounts (<0.5%). Hence, “contaminants” will be synonymously used to describe rRNA. After contaminant filtering, the remaining reads were mapped to the genome using STAR aligner using the following parameters: *--outFilterMismatchNoverLmax 0.1 --alignIntronMax 8000 --outSAMmultNmax 1*. Coverage profiles along the genome, or rRNA were generated using bedtools and plotted using the Sushi package in R (Phanstiel, 2022). Mapped reads were counted over annotated CDS using FeatureCounts (Liao et al., 2014) using the parameters: *-M -s 1 -t CDS -g gene_id*. TMM normalization was performed using edgeR (Robinson et al., 2009), and genes with fewer than 10 counts were removed. P-site estimation, metagene analysis, and phase analysis were performed using the “plastid” package (Dunn and Weissman, 2016), and plotted in R. In order to quantify RPF density in each of the genomic features (CDS, 5’UTR, 3’UTR, introns, and intergenic regions), the Arabidopsis GTF file was processed using the “cs generate” function of “plastid” (Dunn and Weissman, 2016) to collapse multiple transcript isoforms into a single transcript isoform. This merged annotation file was used to create individual BED files that describe each of the genomic features. Only uniquely mapped RPFs were used for quantification within genomic features, which were extracted from the BAM alignment using samtools (Li et al., 2009). The genomic position and density of all RPF P-sites was calculated using the “make_wiggle” function of “plastid”. Quantification of RPF P-sites that overlap with each of the genomic features, was performed using the “map” function of “bedtools”.

*Nicotiana Tabacum:* There are two available genomes for tobacco, which are both available from solgenomics (https://solgenomics.net/). The most recent version was used for alignment (Edwards et al., 2017) since it is conveniently assembled into chromosomes. However, the Edwards genome assembly does not have annotations for tRNA. Therefore, tRNA sequences were obtained from the Sierro genome annotations (Sierro et al., 2014). The tobacco chloroplast (NC_001879.2) and mitochondrial (NC_006581.1) genomes were obtained from NCBI. Chloroplast and mitochondrial rRNA sequences were extracted from the respective genome annotations. Nuclear rRNA sequences were obtained from NCBI: 26S (AF479172.1), 18S (AJ236016.1), 5.8S (AJ300215.1) and 5S (AJ222659.1). The remainder of the analysis pipeline follows the same structure as what was performed for Arabidopsis. Additional parameters with example scripts can be found in Supplemental file 1.

# References

**Bartholomäus A, Del Campo C, Ignatova Z** (2016) Mapping the non-standardized biases of ribosome profiling. Biol Chem **397**: 23–35

**Berg JA, Belyeu JR, Morgan JT, Ouyang Y, Bott AJ, Quinlan AR, Gertz J, Rutter J** (2020) Xpressyourself: Enhancing, standardizing, and automating ribosome profiling computational analyses yields improved insight into data. PLoS Comput Biol **16**: 1–20

**Calviello L, Mukherjee N, Wyler E, Zauber H, Hirsekorn A, Selbach M, Landthaler M, Obermayer B, Ohler U** (2015) Detecting actively translated open reading frames in ribosome profiling data. Nat Methods **13**: 1–9

**Chen H, Alonso JM, Stepanova AN** (2022) A Ribo-Seq Method to Study Genome-Wide Translational Regulation in Plants.

**Chiu CW, Li YR, Lin CY, Yeh HH, Liu MJ** (2022) Translation initiation landscape profiling reveals hidden open-reading frames required for the pathogenesis of tomato yellow leaf curl Thailand virus. Plant Cell **34**: 1804–1821

**Chotewutmontri P, Barkan A** (2018) Multilevel effects of light on ribosome dynamics in chloroplasts program genome-wide and psbA-specific changes in translation. PLoS Genet **14**: e1007555

**Chotewutmontri P, Barkan A** (2016) Dynamics of Chloroplast Translation during Chloroplast Differentiation in Maize. PLoS Genet **12**: 1–28

**Chotewutmontri P, Stiffler N, Watkins KP, Barkan A** (2018) Ribosome profiling in Maize. **1676**: 165–183

**Choudhary S, Li W, Smith AD** (2020) Accurate detection of short and long active ORFs using Ribo-seq data. Bioinformatics **36**: 2053–2059

**Chung BY, Hardcastle TJ, Jones JD, Irigoyen N, Firth AE, Baulcombe DC, Brierley I** (2015) The use of duplex-specific nuclease in ribosome profiling and a user-friendly software package for Ribo-seq data analysis. Rna **21**: 1731–1745

**Daniel A, Michael R, Chen Wei-Sheng, Danielsson Maxwell, Fennell Timothy, Russ Carsten, Jaffe David, Nusbaum Chad, Andreas G** (2011) Analyzing and minimizing PCR amplification bias in Illumina sequencing libraries. Genome Biol **12**: 1–14

**Dobin A, Davis CA, Schlesinger F, Drenkow J, Zaleski C, Jha S, Batut P, Chaisson M, Gingeras TR** (2013) STAR: Ultrafast universal RNA-seq aligner. Bioinformatics **29**: 15–21

**Douka K, Agapiou M, Birds I, Aspden JL** (2022) Optimization of Ribosome Footprinting Conditions for Ribo-Seq in Human and Drosophila melanogaster Tissue Culture Cells. Front Mol Biosci **8**: 1–12

**Dunn JG, Weissman JS** (2016) Plastid: Nucleotide-resolution analysis of next-generation sequencing and genomics data. BMC Genomics **17**: 1–12

**Edwards KD, Fernandez-Pozo N, Drake-Stowe K, Humphry M, Evans AD, Bombarely A, Allen F, Hurst R, White B, Kernodle SP, et al** (2017) A reference genome for Nicotiana tabacum enables map-based cloning of homeologous loci implicated in nitrogen utilization efficiency. BMC Genomics **18**: 1–14

**Gao Y, Thiele W, Saleh O, Scossa F, Arabi F, Zhang H, Sampathkumar A, Kühn K, Fernie A, Bock R, et al** (2022) Chloroplast translational regulation uncovers nonessential photosynthesis genes as key players in plant cold acclimation. Plant Cell **34**: 2056–2079

**Gawroński P, Jensen PE, Karpiński S, Leister D, Scharff LB** (2018) Pausing of chloroplast ribosomes is induced by multiple features and is linked to the assembly of photosynthetic complexes. Plant Physiol **176**: 2557–2569

**Gerashchenko M V., Gladyshev VN** (2017) Ribonuclease selection for ribosome profiling. Nucleic Acids Res **45**: e6

**Gotsmann VL, Ting MKY, Haase N, Rudorf S, Zoschke R, Willmund F** (2023) Utilizing high resolution ribosome profiling for the global investigation of gene expression in Chlamydomonas reinhardtii. BioRxiv 1–52

**Hornstein N, Torres D, Das Sharma S, Tang G, Canoll P, Sims PA** (2016) Ligation-free ribosome profiling of cell type-specific translation in the brain. Genome Biol **17**: 1–15

**Hsu PY, Calviello L, Wu H-YL, Li F-W, Rothfels CJ, Ohler U, Benfey PN** (2016) Super-Resolution Ribosome Profiling Reveals Novel Translation Events in Arabidopsis. Proc Natl Acad Sci USA **113**: E7126–E7135

**Ingolia NT** (2016) Ribosome Footprint Profiling of Translation throughout the Genome. Cell **165**: 22–33

**Ingolia NT, Ghaemmaghami S, Newman JRS, Weissman J** (2009) Genome-Wide Analysis in Vivo of Translation with. Science (80- ) **1168978**: 218–324

**Juntawong P, Girke T, Bazin J, Bailey-Serres J** (2014) Translational dynamics revealed by genome-wide profiling of ribosome footprints in Arabidopsis. Proc Natl Acad Sci **111**: E203–E212

**Kraus AJ, Brink BG, Siegel TN** (2019) Efficient and specific oligo-based depletion of rRNA. bioRxiv 589622

**Lauria F, Tebaldi T, Bernabò P, Groen EJN, Gillingwater TH, Viero G** (2018) riboWaltz: Optimization of ribosome P-site positioning in ribosome profiling data. PLoS Comput Biol **14**: 1–20

**Lei L, Shi J, Chen J, Zhang M, Sun S, Xie S, Li X, Zeng B, Peng L, Hauck A, et al** (2015) Ribosome profiling reveals dynamic translational landscape in maize seedlings under drought stress. Plant J **84**: 1206–1208

**Li H, Handsaker B, Wysoker A, Fennell T, Ruan J, Homer N, Marth G, Abecasis G, Durbin R** (2009) The sequence alignment/map format and SAMtools. Bioinformatics **25**: 2078–2079

**Liao Y, Smyth GK, Shi W** (2014) FeatureCounts: An efficient general purpose program for assigning sequence reads to genomic features. Bioinformatics **30**: 923–930

**Liu M-J, Wu S-HS-H, Wu J-F, Lin W-D, Wu Y-C, Tsai T-Y, Tsai H-L, Wu S-HS-H** (2013) Translational landscape of photomorphogenic Arabidopsis. Plant Cell **25**: 3699–710

**Lukoszek R, Feist P, Ignatova Z** (2016) Insights into the adaptive response of Arabidopsis thaliana to prolonged thermal stress by ribosomal profiling and RNA-Seq. BMC Plant Biol **16**: 221

**Mahboubi A, Delhomme N, Häggström S, Hanson J** (2021) Small-scale sequencing enables quality assessment of Ribo-Seq data: an example from Arabidopsis cell culture. Plant Methods **17**: 1–10

**Martin M** (2011) Cutadapt removes adapter sequences from high-throughput sequencing reads. EMBnet.journal **17**: 10–12

**McGlincy NJ, Ingolia NT** (2017) Transcriptome-wide measurement of translation by ribosome profiling. Methods. doi: 10.1016/j.ymeth.2017.05.028

**Merchante C, Brumos J, Yun J, Hu Q, Spencer KR, Enríquez P, Binder BM, Heber S, Stepanova AN, Alonso JM** (2015) Gene-Specific Translation Regulation Mediated by the Hormone-Signaling Molecule EIN2. Cell **163**: 684–697

**Mohammad F, Green R, Buskirk AR** (2019) A systematically-revised ribosome profiling method for bacteria reveals pauses at single-codon resolution. Elife **8**: 1–25

**Murashige T, Skoog F** (1962) A Revised Medium for Rapid Growth and Bio Assays with Tobacco Tissue Cultures. Physiol Plant **15**: 473–497

**Phanstiel DH** (2022) Sushi: Tools for visualizing genomics data. R Packag. version 1.34.0

**Quinlan AR, Hall IM** (2010) BEDTools: A flexible suite of utilities for comparing genomic features. Bioinformatics **26**: 841–842

**Raj A, Wang SH, Shim H, Harpak A, Li YI, Engelmann B, Stephens M, Gilad Y, Pritchard JK** (2016) Thousands of novel translated open reading frames in humans inferred by ribosome footprint profiling. Elife **5**: 1–24

**Robinson MD, McCarthy DJ, Smyth GK** (2009) edgeR: A Bioconductor package for differential expression analysis of digital gene expression data. Bioinformatics **26**: 139–140

**Scharff LB, Ehrnthaler M, Janowski M, Childs LH, Hasse C, Gremmels J, Ruf S, Zoschke R, Bock R** (2017) Shine-dalgarno sequences play an essential role in the translation of plastid mRNAs in Tobacco. Plant Cell **29**: 3085–3101

**Schuster M, Gao Y, Schöttler MA, Bock R, Zoschke R** (2020) Limited responsiveness of chloroplast gene expression during acclimation to high light in tobacco. Plant Physiol **182**: 424–435

**Sierro N, Battey JND, Ouadi S, Bakaher N, Bovet L, Willig A, Goepfert S, Peitsch MC, Ivanov N V.** (2014) The tobacco genome sequence and its comparison with those of tomato and potato. Nat Commun **5**: 1–9

**Smirnova J, Loerke J, Kleinau G, Schmidt A, Bürger J, Meyer EH, Mielke T, Scheerer P, Bock R, Spahn CMT, et al** (2023) Structure of the actively translating plant 80S ribosome at 2 . 2 Å resolution. Nat plants. doi: 10.1038/s41477-023-01407-y

**Trösch R, Barahimipour R, Gao Y, Badillo-Corona JA, Gotsmann VL, Zimmer D, Mühlhaus T, Zoschke R, Willmund F** (2018) Commonalities and differences of chloroplast translation in a green alga and land plants. Nat Plants **4**: 564–575

**Wu CC-C, Zinshteyn B, Wehner KA, Green R** (2019a) High-Resolution Ribosome Profiling Defines Discrete Ribosome Elongation States and Translational Regulation during Cellular Stress. Mol Cell **0**: 1–12

**Wu HYL, Song G, Walley JW, Hsu PY** (2019b) The tomato translational landscape revealed by transcriptome assembly and ribosome profiling. Plant Physiol **181**: 367–380

**Xiao Z, Huang R, Xing X, Chen Y, Deng H, Yang X** (2018) De novo annotation and characterization of the translatome with ribosome profiling data. Nucleic Acids Res **46**: e61

**Xu Z, Hu L, Shi B, Geng S, Xu L, Wang D, Lu ZJ** (2018) Ribosome elongating footprints denoised by wavelet transform comprehensively characterize dynamic cellular translation events. Nucleic Acids Res. doi: 10.1093/nar/gky533

**Yang X, Cui J, Song B, Yu Y, Mo B, Liu L** (2020) Construction of High-Quality Rice Ribosome Footprint Library. Front Plant Sci **11**: 1–16

**Yang X, Song B, Cui J, Wang L, Wang S, Luo L, Gao L, Mo B, Yu Y, Liu L** (2021) Comparative ribosome profiling reveals distinct translational landscapes of salt-sensitive and -tolerant rice. BMC Genomics **22**: 1–17

**Yusupova G, Yusupov M** (2017) Crystal structure of eukaryotic ribosome and its complexes with inhibitors. Philos Trans R Soc B Biol Sci. doi: 10.1098/rstb.2016.0184

**Zinshteyn B, Wangen JR, Hua B, Green R** (2020) Nuclease-mediated depletion biases in ribosome footprint profiling libraries. RNA **26**: 1481–1488

**Zoschke R, Barkan A** (2015) Genome-wide analysis of thylakoid-bound ribosomes in maize reveals principles of cotranslational targeting to the thylakoid membrane. Proc Natl Acad Sci **112**: E1678–E1687

**Zoschke R, Watkins KP, Barkan A** (2013) A Rapid Ribosome Profiling Method Elucidates Chloroplast Ribosome Behavior in Vivo. Plant Cell **25**: 2265–2275
